# Population Structure and Genetic Diversity of Two-Rowed Barley Accessions from Kazakhstan Based on SNP Genotyping Data

**DOI:** 10.3390/plants10102025

**Published:** 2021-09-27

**Authors:** Shyryn Almerekova, Yuliya Genievskaya, Saule Abugalieva, Kazuhiro Sato, Yerlan Turuspekov

**Affiliations:** 1 Laboratory of Molecular Genetics, Institute of Plant Biology and Biotechnology, Almaty 050040, Kazakhstan; almerekovakz@gmail.com (S.A.); julia.genievskaya@gmail.com (Y.G.); absaule@yahoo.com (S.A.); 2 Faculty of Biology and Biotechnology, al-Farabi Kazakh National University, Almaty 050038, Kazakhstan; 3 Institute of Plant Science and Resources, Okayama University, Kurashiki 710-0046, Japan; kazsato@rib.okayama-u.ac.jp

**Keywords:** barley, genetic diversity, population structure, genetic geography, single-nucleotide polymorphism, Illumina Infinium assay

## Abstract

The genetic relationship and population structure of two-rowed barley accessions from Kazakhstan were assessed using single-nucleotide polymorphism (SNP) markers. Two different approaches were employed in the analysis: (1) the accessions from Kazakhstan were compared with barley samples from six different regions around the world using 1955 polymorphic SNPs, and (2) 94 accessions collected from six breeding programs from Kazakhstan were studied using 5636 polymorphic SNPs using a 9K Illumina Infinium assay. In the first approach, the neighbor-joining tree showed that the majority of the accessions from Kazakhstan were grouped in a separate subcluster with a common ancestral node; there was a sister subcluster that comprised mainly barley samples that originated in Europe. The Pearson’s correlation analysis suggested that Kazakh accessions were genetically close to samples from Africa and Europe. In the second approach, the application of the STRUCTURE package using 5636 polymorphic SNPs suggested that Kazakh barley samples consisted of five subclusters in three major clusters. The principal coordinate analysis plot showed that, among six breeding origins in Kazakhstan, the Krasnovodopad (KV) and Karaganda (KA) samples were the most distant groups. The assessment of the pedigrees in the KV and KA samples showed that the hybridization schemes in these breeding stations heavily used accessions from Ethiopia and Ukraine, respectively. The comparative analysis of the KV and KA samples allowed us to identify 214 SNPs with opposite allele frequencies that were tightly linked to 60 genes/gene blocks associated with plant adaptation traits, such as the heading date and plant height. The identified SNP markers can be efficiently used in studies of barley adaptation and deployed in breeding projects to develop new competitive cultivars.

## 1. Introduction

Barley (*Hordeum vulgare* L.) was one of the first domesticated cereal crops, showing a dramatic adaptation to various climates and environmental conditions across a wide geographic range and being widely cultivated in all temperate regions. It ranks as the fourth most important cereal crop globally, after wheat, maize, and rice, in terms of planting areas and production and is mainly utilized for animal feed, brewing malts, and human food [1]. In Kazakhstan, the largest Central Asian country, barley is the second most important cereal commodity after wheat, with an average annual total grain yield of 2 million tons [2]. The production of barley has significantly dropped since the Soviet Union era, as the cultivated area has decreased from 7 to 1.5 million hectares. In addition, abiotic [3,4] and biotic [5] stresses cause significant yield losses. Despite this, barley is cultivated across a wide range of eco-geographical niches, and the country is one of the top barley exporters in the world [1].

Cultivated barley, *Hordeum vulgare* L. subsp. *vulgare* (*H. vulgare*), is descended from the wild progenitor *Hordeum vulgare* L. *subsp*. *spontaneum* (C. Koch) Thell (*H. spontaneum*). *H. vulgare* originated 5.5 million years ago in southwest Asia and was disseminated in the Eastern Mediterranean, the Balkans, North Africa, Central Asia, and Tibet [6,7,8,9]. N. Vavilov [10] noted two primary centers of barley origin—the Mediterranean Center and the Abyssinian (now Ethiopian) Center. The first evidence of barley cultivation was uncovered in archaeological excavations in the Fertile Crescent, which date back to ca. 10,000 BP [11]. The Fertile Crescent is well known as a primary center of barley origin, diversity, and domestication. However, its isolated populations spread as far as North Africa, the European shores of the Mediterranean Basin, and East Asia [6]. The polyphyletic domestication of barley took place in at least three centers: the Fertile Crescent, Central Asia, and Tibet [8,9,12,13].

The modern approaches for assessing a crop’s contemporary phylogeny, including that of barley, mainly rely on the availability of diverse germplasm collections at centralized genebanks and the development of new-generation high-throughput genotyping tools. There are a number of genebanks around the world that store barley genetic resources. The various barley collections include modern cultivars, landraces, wild relatives, genetic and cytogenetic stocks, and breeding lines. There are several major seed collection holders of *Hordeum* (barley), including: (1) PGRC—Plant Gene Resources of Canada [14]; (2) NSGC—The USDA-ARS National Small Grains Collection [15,16]; (3) CENARGEN—Embrapa Genetic Resources and Biotechnology: Embrapa Cenargen [17]; (4) ICARDA—International Center for Agricultural Research in the Dry Areas [18]; (5) NIAS—The National Institute of Agrobiological Sciences, reorganized to the National Agriculture and Food Research Organisation (NARO) in 2016 [19]; (6) IPK—the Leibniz Institute of Plant Genetics and Crop Plant Research [20,21,22]; and (7) the Vavilov Institute of Plant Industry (VIR, Russia), which holds more than 20,000 barley accessions [23]. In addition, there are well-established repositories and data-deposition and data-exchange formats for genomic data, such as the National Center for Biotechnology Information (NCBI) [24], the European Nucleotide Archive (ENA) [25], and the DNA Data Bank of Japan (DDBJ) [26], which are part of the International Nucleotide Sequence Database Collaboration [27].

The release of the draft barley (*Hordeum vulgare*) genome (International Barley Genome Sequencing Consortium (IBSC) [28] and the development of widely accepted and accessible single-nucleotide polymorphism (SNP) arrays led to the massive high-throughput genotyping of large-size collections. The development of new-generation genotyping tools started from the development of early oligonucleotide pooled assays with several thousand SNPs [29,30] that later expanded to 50,000 SNP Illumina arrays [31,32]. In addition, many other high-throughput genotyping tools were developed, such as DArTseq [33], restriction-site-associated DNA sequencing (RAD-Seq) [34], genotyping-by-sequencing [35], and RNA-seq [36] technologies. Most of these technologies were instantly applied to population structure and worldwide barley phylogenetic studies using *H. spontaneum* and *H. vulgare* collections [22,37,38,39,40]. For instance, Bengtsson et al. [38] and Elakhdar et al. [39] demonstrated the importance of the high-throughput DNA genotyping of barley accessions for the assessment of population structure in regional collections [38,39]. Milner et al. [22] analyzed genome-wide GBS data for barley accessions from the German national genebank and assessed the global population structure of domesticated barley. In addition, the authors detected known and novel loci underlying certain morphological traits that differentiate barley gene pools. They underlined the importance of genomic tools for genebank management and the efficient utilization of germplasm collections [22]. Based on the diversity analyses of Milner et al. [22], the global landscape of the barley genome [41,42] was recently analyzed using 20 cultivated and wild accessions [43]. Hill et al. [40] demonstrated the contributions of both historical and recent breeding efforts to local adaptation and crop improvement in a global barley panel by analyzing the distribution of genetic variants with respect to a geographic region or historical breeding category.

Despite the importance of Kazakhstan for the worldwide barley market and the specificity of geographic locations in the 9th largest country in the world, none of the aforementioned global population structure studies involved Kazakh accessions in their research [22,40]. This may be due to the low-level integration of Kazakh barley research organizations and scientists in the mainstream international barley community, the restricted germplasm exchange with major genebank holders, and the limited applications of new genotyping tools in genetic and breeding studies. Despite this, several accessions of landraces and old and replaced cultivars from Kazakhstan that have been assessed in large-scale studies using simple sequence repeat (SSR) markers are available at the VIR (Russian Federation) genebank [44]. Moreover, the population structure of wild barley accessions from Southern Kazakhstan [45] was determined. Genome-wide association studies (GWAS) for the identification of quantitative trait loci (QTLs) of agronomic traits using 92 cultivars and promising lines of cultivated barley from Kazakhstan [3,5] were performed using SNP Illumina arrays. However, additional studies are required to analyze the comparative distribution of genome-wide variations in modern cultivars with respect to geographic regions of cultivation both within the country and worldwide. Ultimately, these types of studies may enhance breeding efforts that promote a better understanding of plant adaptation to local environments and, eventually, the construction of new competitive cultivars. Studies of this type have become increasingly feasible with the availability of open-source curated databases, such as the SNP database from the James Hutton Institute at the Germinate Barley SNP Platforms (https://ics.hutton.ac.uk, accessed date 16 March 2021) [46]. Therefore, the purposes of this work were to study the population structure and estimate the genetic diversity in collections of local modern two-rowed barley cultivars from Kazakhstan and accessions from diverse geographic regions around the world, to assess the local and global population structure using SNP data, and to identify key SNP markers and genes involved in the adaptation processes of barley.

## 2. Results

### 2.1. The Relationship of Two- and Six-Rowed Barley Accessions Based on SNP Marker Analysis

**Figure 1:**
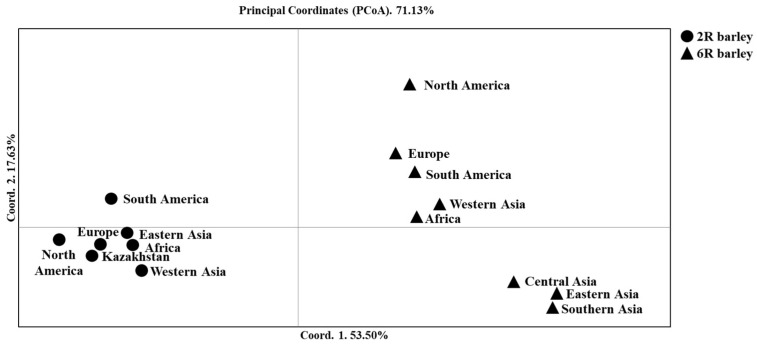
The principal coordinate analysis (PCoA) of the barley collection using 1955 polymorphic SNP markers and Nei’s unbiased genetic distance.

The collection consisting of 597 two-rowed and 798 six-rowed modern barley accessions was analyzed using 1955 polymorphic SNP markers representing all seven chromosomes of the genome. The number of polymorphic SNP markers analyzed varied from 225 SNPs on chromosome 1H to 357 SNPs on chromosome 5H. The neighbor-joining (NJ) tree (Supplementary Figure S1) and PCoA (Figure 1) are clearly separated accessions into two- and six-rowed barley groups. The PCoA study further separated seven two-rowed and eight six-rowed groups of accessions based on their origins (Figure 1). The first principal coordinate elucidated 53.5% of 71.1% of the total variation and divided the accessions according to their row type, and the second principal coordinate (17.6%) further separated the groups of six-rowed barley accessions with different origins. The six-rowed accessions from Central, East, and South Asia were placed in the right bottom box, while accessions from the remaining five origin groups with the same row type were positioned in the right top box of the plot (Figure 1). The Nei unbiased distance index, similar to the PCoA plot, indicated that six-rowed accessions from Africa (0.145) and West Asia (0.149) are the closest to the two-rowed accessions from West Asia (Supplementary Table S1), which is the region in which domesticated barley originated [47].

### 2.2. The Clusterization Analysis of Accessions from Kazakhstan in the Two-Rowed Barley Collection

**Figure 2:**
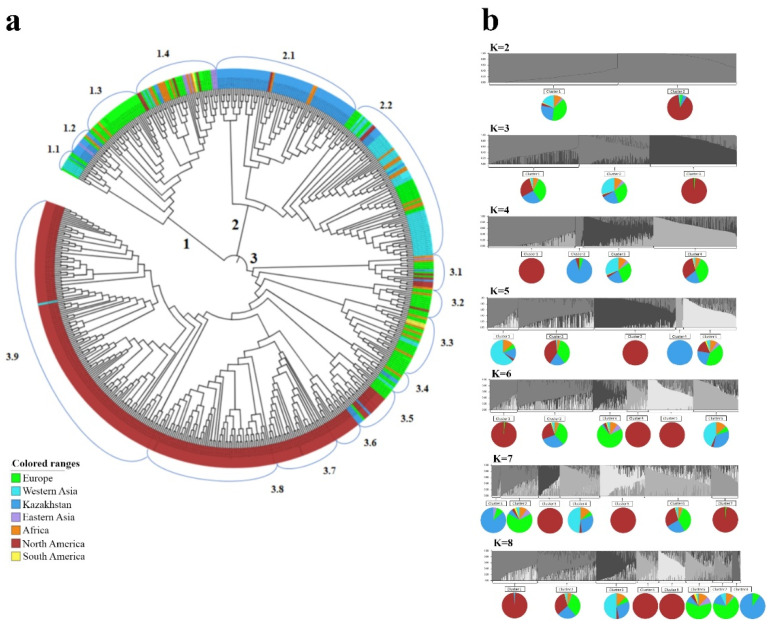
The neighbor-joining tree and population structure of the two-rowed barley collection. (**a**) The clusterization using 597 accessions from seven different barley regions around the world and 1955 single-nucleotide polymorphism (SNP) markers. (**b**) Graphical representation of the clusterization for two-rowed barley accessions using the STRUCTURE package and K2-K8 steps.

The NJ trees of two-rowed accessions were constructed using 597 samples from seven different regions of the world and 1955 SNPs, including 94 samples from Kazakhstan, using 5636 SNPs. The study of 597 samples revealed that the majority of accessions from Kazakhstan were grouped in Subclusters 2.1 and 1.2 (Figure 2a). The accessions from West Asia, Africa, and Europe were well spread in all three main clusters. The accessions from East Asia were mostly located in Cluster 1, while barley from the USA, the largest group in the collection (279 accessions), mostly occupied Cluster 3 (Figure 2a). The largest unbiased diversity index (0.314) was recorded for the accessions from Africa, followed by those from East Asia (0.313) and Europe (0.309) (Table 1).

**Figure 3:**
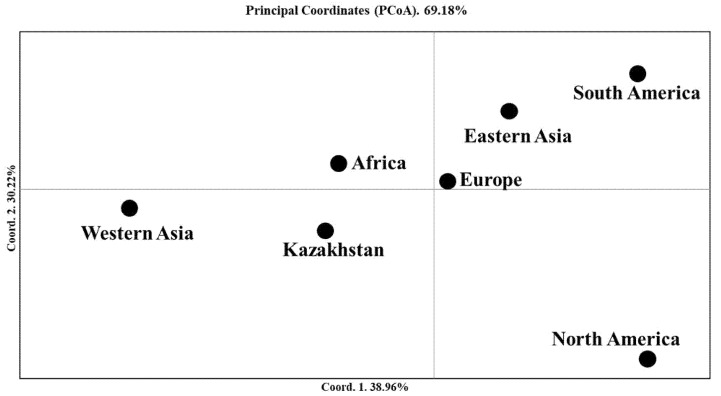
Principal coordinate analysis (PCoA) of seven groups of two-rowed barley accessions with different breeding origins using 1955 polymorphic SNP markers and Nei’s unbiased distance index.

The PCoA of seven groups for two-rowed barley accessions (Figure 3) was very similar to the left part in the plot shown in Figure 1. However, Figure 3 provided several additional patterns that helped to explain the relationship between the breeding origins of the studied groups. The first principal coordinate (39.0%) suggested that samples from Kazakhstan are close to samples from Africa and Europe, while samples from Western Asia were the most distant from those from South and North America. The second principal coordinate 2 (30.2%) enabled a good differentiation between North America and South America, East Asia, and Europe (Figure 3). Nei’s unbiased distance index suggested that African samples were the most genetically similar to samples from West Asia (Table 2).

In addition, the subdivision of the two-rowed barley collection was studied using the STRUCTURE package with a range of steps from K = 2 to 10 (Supplementary Table S2). At the K = 2 Step, most accessions were in Cluster 1, while Cluster 2 was populated by 269 samples from the USA, 17 from Kazakhstan, 9 from Europe, and 1 from Africa. This indicates the specificity of US barley, as only 10 samples were assigned to Cluster 1. At the K = 4 Stage, the majority of the samples were grouped in Clusters 3 and 4, while Cluster 1 was heavily populated by samples from the USA (209 out of 210), and Cluster 2, by samples from Kazakhstan (19 out of 21). The data assessment in the remaining K Steps (from K = 5 to 10) did not significantly change the structural composition. Notably, at the K = 8 Step, Cluster 4 at K = 4 was separated into three more clusters (Clusters 3, 6, and 7), and the USA Cluster 1 at Stage 4 was split into three more clusters (Clusters 1, 4, and 5) (Supplementary Table S2). The Evanno [48] test of the Structure Harvester [49] using the “elbow method” conceptualized by R. Thorndike [50] indicated that the optimal number of subpopulations at which the values stabilized was K = 4 (Supplemental Table S2).

The assessment of the relationship between the seven groups from different regions with different breeding origins using SNPs on seven individual chromosomes indicated that the total PCoA index varied from 70.8% on 4H to 88.5% on 6H. The analysis of the PCoA plots for each chromosome (from 1H to 7H) showed a different relationship between the breeding origin groups (Supplementary Figure S2). Particularly, the East Asia group was distinct in the PCoA based on the SNPs on chromosome 3H, the South American group was very distinct on chromosome 7H, and the North American group was very distinct on chromosomes 1H, 2H, 4H, and 5H. Notably, the Kazakh barley was very distinct in the PCoA based on SNPs on chromosomes 2H, 4H, and 6H (Supplementary Figure S2). Arguably, the SNP markers on these three chromosomes (2H, 4H, and 6H) reflect the directions of local breeding programs and may play an important role in the adaptation to Kazakh environments. Therefore, the allele frequencies of the SNPs in these three chromosomes in local accessions were compared with the allele frequencies of the accessions from Africa and Europe that were the most genetically close to the Kazak group (Table 1). A list of SNPs with more than 50% opposite allele frequencies was selected for two case studies; (1) Kazakhstan and Africa (18 SNPs), and (2) Kazakhstan and Europe (23 SNPs) (Supplementary Tables S3 and S4). As the alleles in these SNPs were dominated with frequencies higher than 50% in samples of the Kazakhstan group, they had frequencies of less than 50% in accessions of African and European groups. The comparison of the genetic positions of SNPs in both studies showed several tight linkages of these markers with genes associated with row type, heading time, and plant height, as analyzed in studies by Alqudah et al. [51,52]. For instance, the SNPs on chromosome 2H were tightly linked with *eps2/HvHOX2/HvGID2/HvKNOX1/HvD11/HvCEN* genes in both pairwise assessments, the *HvSSIIIb* gene in the Kazakhstan vs. Africa case, and the *HvMAX3/HvCCD7/HvHTD1/HvD17* gene in the Kazakhstan vs. Europe case. On chromosome 4H, the identified SNPs were positioned in the vicinity of the genes *HvCO16/HvPRR59/HvPhyb* in the Kazakhstan vs. Africa case, and the gene *HvHTD2* in the Kazakhstan vs. Europe case. On chromosome 6H, the linkages of BOPA1_3658-1310 with *HvCO5* in the Kazakhstan vs. Africa case and 11_10015 with *HvTPS2* in the Kazakhstan vs. Europe case were also noted (Supplementary Table S4).

**Figure 4:**
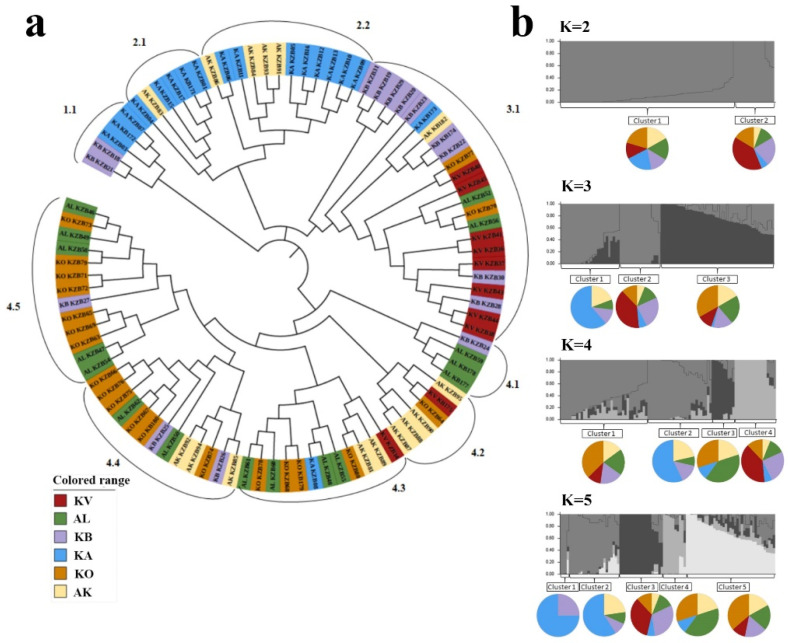
The neighbor-joining tree and population structure of the two-rowed barley collection from Kazakhstan. (**a**) The clusterization using 94 accessions from six breeding programs in Kazakhstan using 5636 SNPs. (**b**) Graphical representation of the clusterization for barley accessions from Kazakhstan using the STRUCTURE package at Steps K2-K5. See the abbreviations of breeding stations in the main text.

The assessment of the clusterization of 94 Kazakh samples using 5636 SNPs suggested the separation of accessions into four clusters (Figure 4a). The most populated group was Cluster 4 (51.1%), followed by Cluster 3 (24.5%), Cluster 2 (19.1%), and Cluster 1 (5.3%). The NJ tree indicated that samples from KA dominated in the 1.1, 2.1, and 2.2 subclusters; samples from KB and KV dominated in subcluster 3.1; and samples from KO, AL, and AK were heavily present in the majority of the groups of Cluster 4 (Figure 4a).

### 2.3. The Genetic Geography of Two-Rowed Barley Accessions from Six Breeding Programs of Kazakhstan According to the Illumina SNP Array

**Figure 5:**
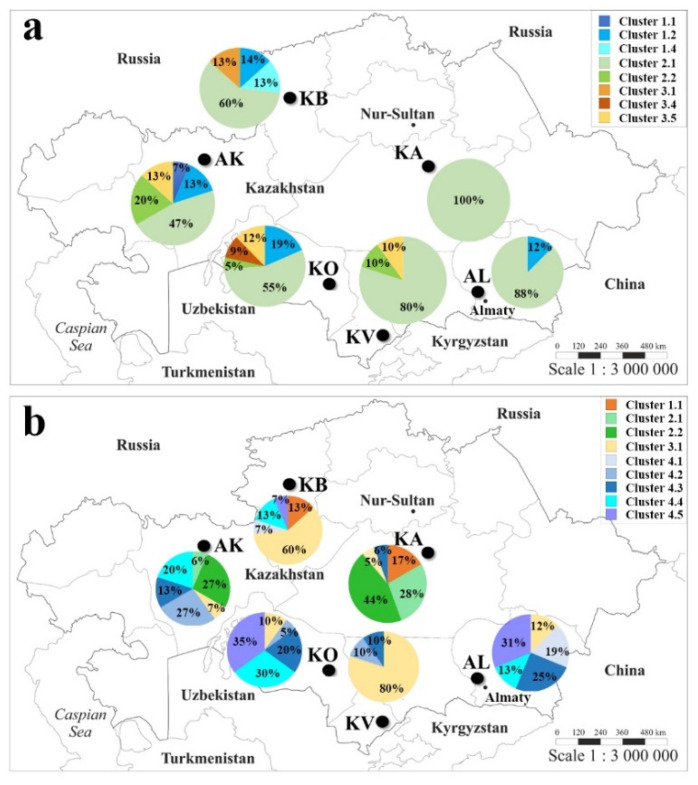
Genetic geography of two-rowed barley from Kazakhstan according to neighbor-joining (NJ) clusterization. (**a**) Characterization based on the NJ tree of the world barley collection using 1955 SNP (single-nucleotide polymorphism) markers. (**b**) Characterization based on the NJ tree using 5636 SNPs. See the abbreviations of breeding stations in the main text.

The assignment of cluster groups and subgroups for the 94 studied accessions allowed for the characterization of the composition of each of the six breeding programs in Kazakhstan using 1955 SNPs (Figure 5a) and 5636 SNPs (Figure 5b). The NJ clusterization using 1955 SNPs helped us to assess the relationship between local accessions and samples from other regions of the world, and the clusterization using 5636 SNPs facilitated the detailed grouping of accessions within the country using a significantly larger number of markers.

As stated above, the clusterization of two-rowed barley in Figure 2a suggests that Kazakh barley is mostly represented in Subclusters 2.1 and 1.2. In particular, the partitioning of accessions from six groups of different origins indicated that most of the samples in a local breeding program were located in Subcluster 2.1 (Figure 2a). The accessions in Subcluster 2.1 were heavily present in Karaganda (KA, Central Kazakhstan; 100%), followed by the Almaty (AL, South-East Kazakhstan; 88%) and Krasnovodopad (KV, South Kazakhstan; 80%) breeding programs. The breeding programs in environmentally stressful sites, such as Aktobe (AK, West Kazakhstan; mostly affected by drought stress) and Kyzylorda (KO, South Kazakhstan; mostly affected by drought and high-soil-salinity stress), consisted of samples from six and five different subclusters, respectively (Figure 5a). The breeding pool at the Karabalyk (KB, North Kazakhstan), the region that represents more than 80% of the Kazakhstan harvesting area, was represented by 60% of the samples in Cluster 2, 27% of the samples in Cluster 1, and 13% of the samples in Cluster 3 (Figure 5a).

The assessment of the clusterization of local accessions using 5636 SNPs further clarified the patterns in genetic geography in Kazakhstan (Figure 5b). It became apparent that the samples in Cluster 1 in the NJ tree (Figure 4a) were minorities and were only found at the KB (13%) and KA (17%) programs. The samples in Cluster 2 prevailed at the KA program (72%) and were moderately present at the AK program (33%). The samples in Cluster 3 were distributed throughout the programs and dominated at the KV (80%) and KB (60%) programs. Finally, the samples in Cluster 4 were the most populous group and dominated at the KO (90%), AL (88%), and AK (60%) programs (Figure 5b).

**Figure 6:**
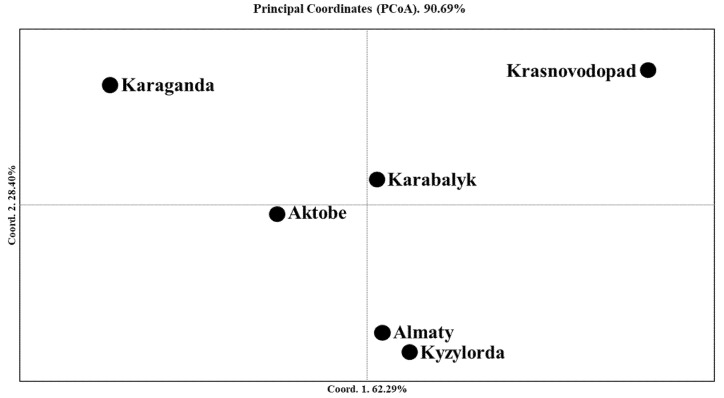
Principal coordinate analysis (PCoA) of six barley groups from different geographic regions of Kazakhstan using 5636 polymorphic SNPs and Nei’s unbiased distance index.

The PCoA suggested that the closest genetic similarities existed between the Almaty and Kyzylorda stations, while PC1 showed that the accessions in the Krasnovodopad and Karaganda breeding stations were the most distant, as compared to the other origin groups (Figure 6 and Table 3). The greatest Nei’s unbiased genetic diversity was recorded for the Kyzylorda (0.355), followed by the Aktobe (0.341) and Almaty (0.322) breeding programs (Table 3).

The STRUCTURE analysis using 94 accessions of Kazakhstan and 5636 polymorphic SNP markers at the K = 2 step clearly separated the KV samples from samples from other breeding programs, as 80% of the KV accessions were in Cluster 2, while 77.7% of samples from the other five origins were in Cluster 1 (Supplementary Table S5). At Step K3, the other major separation from Cluster 1 at Step K = 2 is visible for samples from KA, where 88.9% of the accessions formed a separate cluster. Assessments of the population structure at Steps K = 4 and K = 5 revealed two more minor groups that did not change the major partitioning of samples at Step K = 3 (Supplementary Table S5). The Evanno assessment of the STRUCTURE Harvester for two-rowed barley accessions from Kazakhstan indicates that the optimal number of clusters is two and that this becomes stabilized at the K = 5 Step.

In order to elucidate the most important genetic factors associated with plant adaptation within Kazakhstan, the accessions from the Krasnovodopad breeding program (KV, Southern Kazakhstan) were analyzed against accessions from Karaganda (KA, Central Kazakhstan) and Karabalyk (KB, Northern Kazakhstan), using SNP markers with at least 65% opposite allele frequencies. In these cases, the alleles of selected SNPs were dominated in KV with a frequency higher than 65%, while in KB and KA the same alleles were having less than 35%. In Comparison 1, KV accessions were analyzed against KA accessions, as these groups were the most genetically distant from each other (Figure 6). Initially, 753 SNPs were extracted with selected criteria for opposite allele dominance between these two sites. The genetic positions of these 753 SNPs in all seven chromosomes were compared to the identified genes associated with the heading time and plant height reported by Alqudah et al. [51,52]. The comparison allowed us to identify 214 SNP markers that were tightly linked with 60 known genes or gene blocks (<2.5 cM) that regulate optimal plant adaptation to different geographic regions (Supplementary Table S3). The list of these associations included SNP markers that were in LD with the genes *Vrn-H1* (5H), *Vrn-H2* (4H), *HvFT2* (3H), *HvFT4* (2H), *Ppd-H1* (2H), and *Ppd-H2* (1H) and other important genetic factors affecting flowering time (Supplementary Table S4). The remaining SNPs that are unlinked with those 60 genes/gene blocks are potentially very important DNA marker sources for the additional search of genes involved in the plant adaptation process. In Comparison 2, the KV accessions were analyzed against KB accessions, as the latter group represents the region in which more than 80% of barley is cultivated in Kazakhstan. In this comparison, 118 SNPs out of 5636 available polymorphic markers were selected using the same criteria as in Comparison 1 (Supplementary Table S3). As in the previous case, the genetic positions of these 118 SNPs were compared to the genes listed in the study by Alqudah et al. [51]. The result suggested that 40 SNP markers were in tight linkage with 28 known genes that regulate the optimal plant adaptation to different geographic regions (Supplementary Figure S3 and Supplementary Table S4). The analysis of Supplementary Table S4 showed that 25 out of 60 genes/gene blocks were common between the KV/KA and KV/KB comparisons. The identified SNPs in both comparisons (KV/KA and KV/KB) were aligned with SNPs in the markers’ list with opposite allele dominance between Kazakhstan and Africa and between Kazakhstan and Europe (Supplementary Table S4). The results of the alignment suggested that twelve genetic factors were common in both intraregional and large-scale interregional studies (Supplementary Table S3), including six genes/gene blocks on chromosome 2H, two on chromosome 4H, and four on chromosome 6H. Particularly, the list of genetic factors included *HvFT4* and the gene blocks *eps2/HvHOX2/HvGID2/HvKNOX1* on chromosome 2H, *HvPRR59/HvPhyB/HvPRR73/HvGELP112/HvWDL1* on chromosome 4H, and the genes *HvCO4*, *HvCO5*, *HvCO11*, *HvCO14*, and *HvCO16* on chromosomes 2H, 4H, and 6H (Supplementary Figure S3).

## 3. Discussion

Despite Kazakhstan being one of the top barley producers in the world [2], the genetic variability of modern cultivars from Kazakhstan has been rather poorly studied. For instance, in the context of the assessment of global barley diversity, Milner et al. [22] studied the genetic profiles for the barley (*Hordeum vulgare*) collection at the German genebank, which is one of the largest genebanks in the world. Milner et al. (2019) analyzed GBS data from a total of 22,626 DNA samples of barley accessions. The assessment of the Supplementary Table S1, which lists the accessions of Milner et al. [22], suggests that none of the barley samples from Kazakhstan were used in the analysis. Another example is a report by Lister et al. (2018) in which the authors studied the evolutionary pathways of barley in Eurasia using 351 accessions of *H. vulgare*, 142 accessions of *H. spontaneum*, 23 samples of *H. vulgare* f. *agriocrithon* (six rowed brittle rachis barley), and polymorphic SSR markers. The study included several landraces and cultivars from Kazakhstan that were available in the genebank at the N. I. Vavilov Institute (St. Petersburg, Russia). However, in order to better understand the ongoing processes of plant adaption to the constantly changing environments of Kazakhstan, substantially larger sets of modern cultivars and breeding lines that are genotyped using SNP arrays covering whole genomes are required, including comparisons with barley samples from different regions around the world. To this end, the genotyping data comprising 5636 SNPs of 94 modern cultivars and promising lines originating from six different Kazakh breeding programs were collected to determine the population structure and geographic distribution of genetic variants. Moreover, the use of SNP data for the studied collection allowed us to assess the genetic relationship of local accessions with samples from other regions around the world. The resulting NJ tree for 1395 accessions based on 1955 SNPs seems to be robust, as two- and six-rowed barley were clearly separated into two large groups (Supplementary Figure S1), which is in accordance with previously published studies [53,54]. Within the six-rowed barley, the PCoA (Figure 1) showed a distinct difference between the Central, Eastern, and Southern Asian samples, on the one hand, and the other origin groups, on the other, supporting the theory of the genetic differentiation of the occidental and oriental divergence of barley proposed by Takahashi [55].

### 3.1. The Clusterization Analysis of the Two-Rowed Barley Collection

Since breeders in Kazakhstan have traditionally focused on breeding two-rowed spring-type barley, most studies have concentrated on a two-rowed barley germplasm. There are two major concepts concerning the distribution of cultivated barley in the Central Asian region, including Kazakhstan. First, the barley cultivated in Kazakhstan, as part of Central Asia, was spread in the region as a result of the Great Silk Road [44]. Second, the spread of barley was directed from Eastern European territories toward the south, as a result of the barley cultivation activities during the Soviet Union era, when most of the sources for the development of new cultivars were accessions from Ukraine and the Russian Federation [2]. A similar study on the genetic diversity of wheat accessions from Kazakhstan [56], a major cereal crop in the country, suggested that the second assumption might also be a solid hypothesis for barley. Hence, it was assumed that the study of the structure and genetic relationships of modern barley accessions based on a genome-wide SNP analysis might shed light on the relationship of Kazakh accessions with barley samples from other regions around the world. Particularly, the NJ tree that was generated using barley accessions from seven different regions (Figure 2a) indicated that the majority of local samples, including contrasting KA and KV samples (Figure 6), were grouped into Subcluster 2.1. Subcluster 2.1 is a sister clade of Subcluster 2.2, which was dominated by samples from Europe. Therefore, it seems that the second assumption for the genetic distribution of barley samples in Kazakhstan is a more feasible scenario.

The Evanno test using the STRUCTURE Harvester for the entire two-rowed collection suggested that the smallest number of subpopulations at which the values stabilized was K = 4 (Supplementary Table S2). Therefore, in the assessment of the STRUCTURE outputs, the K = 4 subdivisions of samples seem to be most accurate. It appeared that, at this step, the majority of Kazakh accessions (75 samples) were in Clusters 3 and 4, together with accessions from Europe, the USA, Africa, and West Asia. In Clusters 1 and 2, the majority of accessions were from the USA and Kazakhstan, respectively. Hence, the presence of 19 accessions from Kazakhstan (20.2% of the 94 Kazakh samples) in Cluster 2, which consists of 21 accessions, suggested that 1/5 of the local breeding material is genetically distinct from rest of the local population. The PCoA graph and Nei’s genetic distance indices for two-rowed barley indicated that Kazakh samples were the most genetically similar to groups of African, European, and West Asian origin (Figure 3 and Table 2). The results confirmed that these three regions (Western Asia, Africa, and Europe) possess a rich barley genetic variability (Table 1), as was reported in many previous studies [57,58,59]. The close genetic relationship of Kazakh and European barley samples was a predictable result and presumably confirms the strong cooperation in East European breeding programs during the Soviet Union era [2], such as in Ukraine and the Russian Federation. In fact, in the pedigrees of most of the accessions at the KA (Karaganda) station, there were cultivars from Ukraine, including Donetsky 6, Donetsky 9, Donetsky 650, and Odessky 36 (Supplementary Table S6). The close genetic relationship of Kazakh accessions with samples from Africa is possibly associated with breeding activities at the KB (North Kazakhstan) and KV (South Kazakhstan) programs, where local breeders heavily used Ethiopian barley samples acquired from VIR (St. Petersburg, Russia), as these African samples were a source of resistance to different smut diseases [60]. The genetic similarity of samples that originated from KB and KV programs is clearly visible in Figure 2b and Figure 6. Overall, the application of both distance-based (NJ and PCoA) and character-based (Bayesian algorithm in STRUCTURE) methods showed similar results in the clusterization of Kazakhstan barley accessions in comparison to samples from six other regions of the world. Particularly, the distinct cluster 1.2 of Kazakhstan samples in the NJ tree showed precisely the same clusterization of these individuals in the STRUCTURE output at Steps K = 7 and K = 8 (Figure 2b; Supplementary Table S6). At the same time, the application of PCoA and STRUCTURE showed similar outcomes in the clusterization of the group of samples, as both approaches indicated the close genetic relationship between Kazakh and European accessions (Figure 2b and Figure 4). Hence, the application of these methods in the study has efficiently complemented each other.

### 3.2. The Genetic Geography of Kazakhstan Barley Accessions

Kazakhstan is geographically vast, stretching 1652 km from the southern to the northern boundaries. Hence, the intraregional genetic geography of accessions originating in various eco-geographical niches is one of the study’s appealing aspects. A set of 94 cultivars and promising lines from Kazakhstan were analyzed using two different approaches. Firstly, the local collection was analyzed in comparison with accessions from other regions around the world using 1955 polymorphic SNPs (Figure 2a). Secondly, the accessions from six breeding programs within the country were analyzed using 5636 polymorphic SNPs (Figure 4a). According to the latitudes of the locations, these six sites can be separated into two groups: AL, KV, and KO, located below the 45°N parallel, and KB and AK, located above the 45°N parallel [2].

In the first approach, the assessment of the results in Figure 5a indicates that the number of clusters in the Kazakh barley collection varied from one subcluster in the KA region to five subclusters in two stressed environments, AK and KO. Since these two stressed regions are located below and above the 45°N parallel, it appears that there is no strict geographic separation of local barley groups with different origins. On the contrary, the accessions from Subcluster 2.1 dominated in all six of the studied groups (Figure 5a). In the second approach using 5636 SNPs (Figure 5b), the genetic geography map was drawn based on the clusterization in Figure 4a, in which Kazakh accessions were separated into four clusters. However, the accessions in Clusters 1 and 2 appear to be as genetically close as most of the samples from the KA program (Figure 5b), where breeders predominantly used Ukrainian varieties for crosses. The results of the STRUCTURE analysis at the K = 2 Step indicate that eight accessions from the KV are clearly different from the majority of accessions from the other five regions (Supplementary Table S5). At the K = 3 Step, the accessions from the KA station were separated from the major group that was formed at the K = 2 Step. The K = 4 and K = 5 Steps formed another two minor subpopulations that did not significantly change the structure, which was formed at Step K = 3 (Figure 4b; Supplementary Table S5); therefore, it was assumed that groupings of five subclusters in three major clusters correctly reflected the Kazakhstan collection.

### 3.3. The Mining of SNP Markers Associated with Plant Adaptation in Different Regions of Kazakhstan

In order to identify SNP markers that might play an important role in barley’s adaptation to different environments, KV samples from Southern Kazakhstan were compared with KA and KB samples (Central and Northern regions, respectively) using 5636 polymorphic SNP markers. The KV samples were selected for two reasons. First, they grow in the most southern region of the country (Figure 5), and second, the PCoA plot (Figure 6) and STRUCTURE outputs (Supplementary Table S5) suggested that KV samples formed a separate cluster, separate from the majority of Kazakh accessions. The KA accessions were selected because they were the most genetically distant group from KV accessions and formed the separate cluster at the K = 3 Step of the STRUCTURE analysis. The KB accessions were also selected for two reasons. First, the KB accessions are grown in the country’s most northern region, and second, this represents over 85% of the country’s total barley harvesting area. Hence, two different comparisons were used in the mining of SNP markers for plant adaptation in barley. In Comparison 1 (or the KV vs. KA study), 214 SNPs for 60 genes/gene blocks were identified, while in Comparison 2 (the KV vs. KB study), 40 SNPs for 28 genes/gene blocks associated with the heading time and plant height [51,52] were determined (Supplementary Table S4). Similar studies using Kazakh samples vs. European and African samples, which were the most genetically close groups, allowed us to identify SNPs associated with eight and seven genes, respectively (Supplementary Table S4). The smaller number of associations with plant-adaption-related genes in the latter cases was expected, as the number of SNPs in Kazakhstan was nearly three times higher (5636 SNPs) than that in the world barley collections (1955 SNPs). Among the most notable associations identified in the Kazakh population were SNPs tightly linked with *Vrn-H1* (chromosome 5H), *Vrn-H3* (7H), *Ppd-H1* (2H), *Ppd-H2/HvFT3* (1H), *HvFT2* (3H), *HvFT4* (2H), and *eps2/HvHOX2/HvKNOX1* (2H), as all of these genes play crucial roles in flowering time [48,49] (Supplementary Table S4). Interestingly, the SNPs closely linked to *HvFT4* and *eps2/HvHOX2/HvKNOX1*, along with *HvPRR59/HvPhyB* (4H) and *HvTPS2* (6H), were also selected in the Kazakhstan vs. Europe or Kazakhstan vs. Africa analyses, confirming the importance of these genetic factors despite the smaller number of analyzed SNP markers used in the study. Particularly, the *FLOWERING LOCUS T* and *CONSTANS-like* gene families, including *HvFT4*, *HvCO4*, *HvCO5*, *HvCO11*, *HvCO14*, and *HvCO16*, promote flowering under long-day conditions [61,62,63]. *Earliness per se* genes, including *eps2*, which fine-tunes the flowering time in different environments when both the photoperiod and vernalization requirements are met [64], are another genetic factor detected in this comparison. Finally, *HvTPS2*, which is a member of the trehalose-6-phosphate synthase (TPS) genes, plays an essential role in the protection from abiotic stresses [65,66], is an important modulator in plant development and inflorescence architecture [51], and may contribute to yield components, such as the grain number and grain filling [66].

Thus, the DNA genotyping of the barley collection using a large number of accessions and SNP markers tightly linked to known genes associated with plant adaption to different environments may help in breeding programs based on the deployment of specifically selected DNA markers spread throughout the entire genome. The development of sets for these selected SNP markers of adaptation-related genes and their application in breeding projects can efficiently promote the construction of new competitive barley cultivars in various environments.

## 4. Materials and Methods

### 4.1. Barley SNP Genotyping Sources

The SNP data for the studied barley collection was made up of two sets. The first set consisted of 1955 SNP markers from the world collection of 1395 accessions, including two-rowed (597) and six-rowed (798) barley cultivars, breeding lines, and landraces (Supplementary Table S6). The SNP data for this set were acquired from the James Hutton Institute at the Germinate Barley SNP Platforms (629 accessions; https://ics.hutton.ac.uk, accessed date 16 March 2021), from Dr. T. Blake (538 accessions; Montana State University, MT, USA), from National Bioresource Project, Barley, Japan (94 accessions), and from the National Small Grains Collection (NSGC) at the USA National Plant Germplasm System (40 accessions). Except for the samples from the James Hutton Institute, most of the accessions were previously grown in Kazakhstan and analyzed using a genome-wide association study (GWAS) [3,4]. A total of 597 samples of two-rowed barley from North America (279 samples), Europe (123 samples), Western Asia (58 samples), Africa (31 samples), East Asia (9 samples), and South America (3 samples) were used. The six-rowed collections comprised a total of 798 samples, from North America (318 samples), South Asia (115 samples), Europe (106 samples), Africa (89 samples), Western Asia (68 samples), East Asia (67 samples), Central Asia (23 samples), and South America (12 samples) (Supplementary Table S6). The second set of SNP data consisted of 5636 polymorphic SNP markers from 94 two-rowed spring barley samples from Kazakhstan generated from a previous GWAS of agronomic traits [3] using a 9K Illumina Infinium assay. Since breeders in Kazakhstan predominantly use two-rowed barley, none of the six-rowed barley samples from this country were included in the analysis. The Kazakhstan collection consisted of 94 two-rowed cultivars, and promising lines that originated from six experimental stations, including the Aktobe Agricultural Experimental Station (AES) (AK, Aktobe region, West Kazakhstan), Karabalyk AES (KB, Kostanay region, North Kazakhstan), Karaganda AES (KA, Karaganda region, Central Kazakhstan), Kazakh Research Institute of Agriculture and Plant Industry (AL, Almaty region, South-East Kazakhstan), Kazakh Research Institute of Rice-growing (KO, Kyzylorda region, South Kazakhstan), and Krasnovodopad AES (KV, Turkestan region, South Kazakhstan). The location and geographic, soil, and climatic characteristics (Supplementary Figure S4) for these six regions were reported previously [2,3].

The genetic diversity of the collection was studied using SNP genotyping data for two sets (the entire barley collection and the Kazakh collection), and markers with an SNP call rate <0.95 and MAF (minor allele frequency) <0.05 were removed. As a result, 1955 and 5636 polymorphic SNP markers that satisfied the set criteria for the entire barley set and Kazakhstan accessions, respectively, were selected for further analyses.

The pairwise LD between 5636 SNP markers based on their correlations (R^2^) was calculated using the TASSEL v.5.2.53 (Supplementary Figure S5): a Java-based open-source piece of software [67]. To plot the correlation between the pairwise R^2^ and the genetic distance (LD decay plot), the “R” statistical program was used [68] and is presented in Supplementary Figure S5. The average distance at a 0.1 pairwise correlation (r^2^) was 2.5 cM. Therefore, for the comparative assessment of different collection groups using SNP markers with opposite allele dominance linked to known genes associated with plant adaptation traits according to [51,52], the SNP markers located within a range of 0–2.5 cM of known genes were selected.

### 4.2. Statistical Analysis

The neighbor-joining (NJ) tree was constructed with the TASSEL software [67] using a distance-based NJ analysis. Furthermore, it was visualized using the web-based program iTOL [69]. The principal coordinate analysis (PCoA) based on the Nei’s unbiased genetic distance, Nei genetic distance, number of different alleles (Na), number of effective alleles (Ne), Shannon’s information index (I), diversity (h), unbiased diversity (uh), and percentage of polymorphic loci (%P) was performed using GenAlEx 6.5 [70]. The NJ and PCoA analyses were applied for the clusterization of individual accessions and groups of accessions, respectively. The population structure analysis of the barley collections was performed using the Bayesian Markov Chain Monte Carlo (MCMC) algorithm in STRUCTURE [71]. K values of 2 to 10 were tested, the burn-in period was set to 100,000, and the number of MCMC replications after each burn-in was set to 100,000. The selection of the optimal number of subpopulations was performed using the “Evanno test” [48] and the “elbow method” conceptualized by R. Thorndike [50].

## 5. Conclusions

The genetic distribution of barley accessions that originated in six breeding programs of Kazakhstan was assessed using SNP genotyping and the neighbor-joining tree. The neighbor-joining tree and Principal Coordinate analyses of two-rowed barley were constructed using accessions from Kazakhstan and six other regions around the world and 1955 SNP markers from all seven barley chromosomes. The majority of the accessions from Kazakhstan were grouped into separate subclusters with a sister subcluster that mainly consisted of barley samples originating in Europe. Using 1955 SNP markers and the neighbor-joining method, a map of the genetic differentiation of Kazakh barley was constructed. The application of the STRUCTURE package using 5636 polymorphic SNPs indicated that the Kazakh barley samples consisted of five subclusters in three major clusters. The principal coordinate analysis graph demonstrated that, from the six breeding origins in Kazakhstan, the KV and KA samples were the most distant groups. The comparative analysis of the KV and KA samples using 5636 SNP markers allowed us to identify 214 SNPs with opposite allele frequencies, which were tightly linked to 60 genes/gene blocks associated with plant adaptation traits, such as the heading date and plant height. The results of the study can be efficiently used in barley breeding projects that aim to construct competitive cultivars based on SNP markers of genes contributing to the plant adaptation process.

## Figures and Tables

**Table 1:** Genetic diversity in seven groups of two-rowed barley with different breeding origins using 1955 polymorphic SNP markers.

Origin	n	Na	Ne	I	h	uh	%P
Africa	31	1.929 ± 0.006	1.515 ± 0.008	0.460 ± 0.005	0.304 ± 0.004	0.314 ± 0.004	92.94%
East Asia	9	1.747 ± 0.010	1.479 ± 0.008	0.413 ± 0.006	0.278 ± 0.004	0.313 ± 0.005	74.68%
Europe	123	1.966 ± 0.004	1.519 ± 0.008	0.463 ± 0.005	0.306 ± 0.004	0.309 ± 0.004	96.57%
Kazakhstan	94	1.920 ± 0.006	1.445 ± 0.008	0.410 ± 0.005	0.267 ± 0.004	0.270 ± 0.004	91.97%
North America	279	1.925 ± 0.006	1.396 ± 0.008	0.357 ± 0.006	0.233 ± 0.004	0.234 ± 0.004	92.53%
South America	3	1.411 ± 0.011	1.329 ± 0.009	0.262 ± 0.007	0.183 ± 0.005	0.274 ± 0.007	41.07%
Western Asia	58	1.903 ± 0.007	1.453 ± 0.008	0.414 ± 0.005	0.271 ± 0.004	0.276 ± 0.004	90.28%

n = No of samples; Na = No. of different alleles; Ne = No. of effective alleles; I = Shannon’s information index; h = diversity; uh = unbiased diversity; %P = percentage of polymorphic loci.

**Table 2:** Nei’s genetic distances among seven barley groups with different origins.

	Africa	East Asia	Europe	Kazakhstan	North America	South America	Western Asia
Africa							
East Asia	0.027						
Europe	0.014	0.031					
Kazakhstan	0.020	0.052	0.029				
North America	0.068	0.082	0.056	0.072			
South America	0.040	0.045	0.046	0.079	0.087		
Western Asia	0.019	0.078	0.054	0.049	0.110	0.106	

**Table 3:** The average genetic diversity indices of two-rowed barley accessions from six breeding programs in Kazakhstan using 5636 polymorphic SNP markers.

	n	Na	Ne	I	h	uh	%P
Kyzylorda	20	1.932 ± 0.003	1.583 ± 0.004	0.501 ± 0.003	0.337 ± 0.002	0.355 ± 0.002	93.23%
Aktobe	15	1.897 ± 0.004	1.540 ± 0.004	0.474 ± 0.003	0.316 ± 0.002	0.341 ± 0.002	89.72%
Almaty	16	1.853 ± 0.005	1.515 ± 0.005	0.451 ± 0.003	0.302 ± 0.002	0.322 ± 0.002	85.31%
Karabalyk	15	1.868 ± 0.004	1.508 ± 0.005	0.450 ± 0.003	0.299 ± 0.002	0.320 ± 0.002	56.85%
Karaganda	18	1.788 ± 0.005	1.430 ± 0.005	0.388 ± 0.003	0.255 ± 0.002	0.271 ± 0.003	78.82%
Krasnovodopad	10	1.600 ± 0.006	1.173 ± 0.002	0.224 ± 0.003	0.131 ± 0.002	0.145 ± 0.002	60%

n = No of samples; Na = No. of different alleles; Ne = No. of effective alleles; I = Shannon’s information index; h = diversity; uh = unbiased diversity; %P = percentage of polymorphic loci.

## Data Availability

Publicly available datasets that analyzed in this study can be found here: [https://ics.hutton.ac.uk (accessed on 6 August 2021)]. In addition, the data presented in this study are available on request from the corresponding author.

## Supplementary Materials

The following are available online at https://www.mdpi.com/article/10.3390/plants10102025/s1, Figure S1: The neighbor-joining tree of the world collection of barley. Two-rowed barley is highlighted in blue, and six-rowed barley in red. Figure S2: Principal coordinate analysis (PCoA) of seven barley groups with different breeding origins using single-nucleotide polymorphism (SNP) markers on seven individual chromosomes (from 1H to 7H) based on the Nei unbiased distance index. Figure S3: Genetic positions of SNPs with opposite allele frequencies that were associated with specific genes controlling plant adaptation between barley accessions from the Krasnovodopad and Karaganda breeding programs. SNPs linked to the specific genes were selected using a pairwise R^2^ value in the linkage disequilibrium (LD) study (<2.5 cM). Figure S4: Meteorological data for the six experimental stations. (a) The average precipitation (mm) at the six stations for the years 2009–2011. (b) The average mean temperature data (C°) at the six stations in 2009–2011. (c) The locations of the six stations. Figure S5: The pairwise linkage disequilibrium (LD) decay at the critical level of r^2^ = 0.1 using 5636 polymorphic single-nucleotide polymorphism markers of the barley genome. Table S1: Pairwise population matrix of Nei’s unbiased genetic distance for the world barley collection based on 1955 SNP markers. Table S2: The clusterization analysis of two-rowed barley accessions from seven regions of the World using the STRUCTURE package and 1955 SNP markers. (a) The composition of the identified subpopulations of two-rowed barley accessions from seven regions of the world using K2–K10 Steps. (b) Evanno evaluation of the optimal number of sub-populations in two-rowed barley population using a delta K value based on the “Evanno” and “elbow” methods. (c) Graphical representation of the clusterization for two-rowed barley accessions using the STRUCTURE package and K2–K10 Steps. Table S3: The list of identified SNP markers with opposite allele frequencies that were associated with specific genes controlling plant adaptation in the studied barley groups with different breeding origins. List A: South Kazakhstan (KV, Krasnovodopad) vs. Central Kazakhstan origins (KA, Karaganda). List B: South Kazakhstan (KV, Krasnovodopad) vs. North Kazakhstan origins (KB, Karabalyk). List C: Kazakhstan vs. Africa. List D. Kazakhstan vs. Europe. Table S4: Comparative alignment of the identified SNP markers with opposite allele frequencies that were associated with specific genes controlling plant adaptation in studies of accessions with different breeding origins. Table S5: Clusterization of 94 two-rowed barley accessions from Kazakhstan using 5636 SNP markers and the STRUCTURE package. (a) The composition of the identified subpopulations of barley accessions from six regions of Kazakhstan using K2–K5 Steps. (b) Evanno evaluation of the optimal number of sub-populations using a delta K value based on the “Evanno” and “elbow” methods. (c) Graphical representation of the clusterization for barley accessions from Kazakhstan using the STRUCTURE package. Table S6: List of the two- and six-rowed barley accessions that were analyzed in the study using publicly available SNP data.

The following are available online at https://www.mdpi.com/article/10.3390/plants10102025/s1, Figure S1: The neighbor-joining tree of the world collection of barley. Two-rowed barley is highlighted in blue, and six-rowed barley in red. Figure S2: Principal coordinate analysis (PCoA) of seven barley groups with different breeding origins using single-nucleotide polymorphism (SNP) markers on seven individual chromosomes (from 1H to 7H) based on the Nei unbiased distance index. Figure S3: Genetic positions of SNPs with opposite allele frequencies that were associated with specific genes controlling plant adaptation between barley accessions from the Krasnovodopad and Karaganda breeding programs. SNPs linked to the specific genes were selected using a pairwise R2 value in the linkage disequilibrium (LD) study (<2.5 cM). Figure S4: Meteorological data for the six experimental stations. (a) The average precipitation (mm) at the six stations for the years 2009–2011. (b) The average mean temperature data (C°) at the six stations in 2009–2011. (c) The locations of the six stations. Figure S5: The pairwise linkage disequilibrium (LD) decay at the critical level of r2 = 0.1 using 5636 polymorphic single-nucleotide polymorphism markers of the barley genome. Table S1: Pairwise population matrix of Nei’s unbiased genetic distance for the world barley collection based on 1955 SNP markers. Table S2: The clusterization analysis of two-rowed barley accessions from seven regions of the World using the STRUCTURE package and 1955 SNP markers. (a) The composition of the identified subpopulations of two-rowed barley accessions from seven regions of the world using K2–K10 Steps. (b) Evanno evaluation of the optimal number of sub-populations in two-rowed barley population using a delta K value based on the “Evanno” and “elbow” methods. (c) Graphical representation of the clusterization for two-rowed barley accessions using the STRUCTURE package and K2–K10 Steps. Table S3: The list of identified SNP markers with opposite allele frequencies that were associated with specific genes controlling plant adaptation in the studied barley groups with different breeding origins. List A: South Kazakhstan (KV, Krasnovodopad) vs. Central Kazakhstan origins (KA, Karaganda). List B: South Kazakhstan (KV, Krasnovodopad) vs. North Kazakhstan origins (KB, Karabalyk). List C: Kazakhstan vs. Africa. List D. Kazakhstan vs. Europe. Table S4: Comparative alignment of the identified SNP markers with opposite allele frequencies that were associated with specific genes controlling plant adaptation in studies of accessions with different breeding origins. Table S5: Clusterization of 94 two-rowed barley accessions from Kazakhstan using 5636 SNP markers and the STRUCTURE package. (a) The composition of the identified subpopulations of barley accessions from six regions of Kazakhstan using K2–K5 Steps. (b) Evanno evaluation of the optimal number of sub-populations using a delta K value based on the “Evanno” and “elbow” methods. (c) Graphical representation of the clusterization for barley accessions from Kazakhstan using the STRUCTURE package. Table S6: List of the two- and six-rowed barley accessions that were analyzed in the study using publicly available SNP data.
