# Supplementary material for: Population Structure and Genetic Diversity of Two-Rowed Barley Accessions from Kazakhstan Based on SNP Genotyping Data
Source: Plants (Basel). 2021 Sep 27;10(10):2025. doi: 10.3390/plants10102025 (PMC8540147; doi:10.3390/plants10102025)

Figure S2. Principal Coordinates Analysis (PCoA) of seven barley groups with different breeding origins using single nucleotide polymorphism (SNPs) markers in seven individual chromosomes (from 1H to 7H) based on Nei Unbiased Distance index

2R barley, 1H

597 samples (7 groups),
225 SNP

Principal Coordinates Analysis (PCoA)

Pairwise Population Matrix of **Nei Unbiased Genetic Distance**

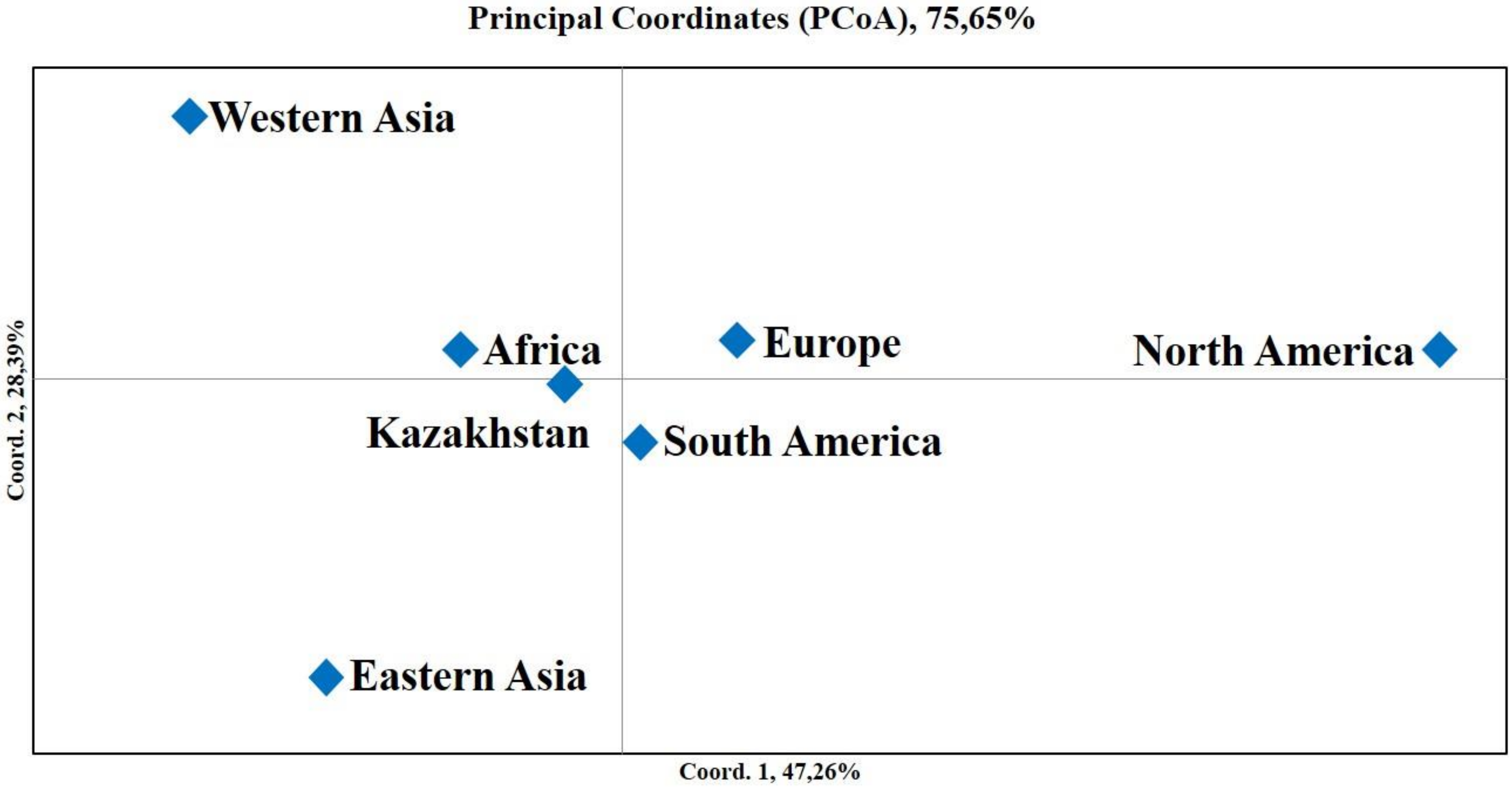

2R barley, 2H

597 samples (7 groups),
296 SNP

Principal Coordinates Analysis (PCoA)

Pairwise Population Matrix of **Nei Unbiased Genetic Distance**

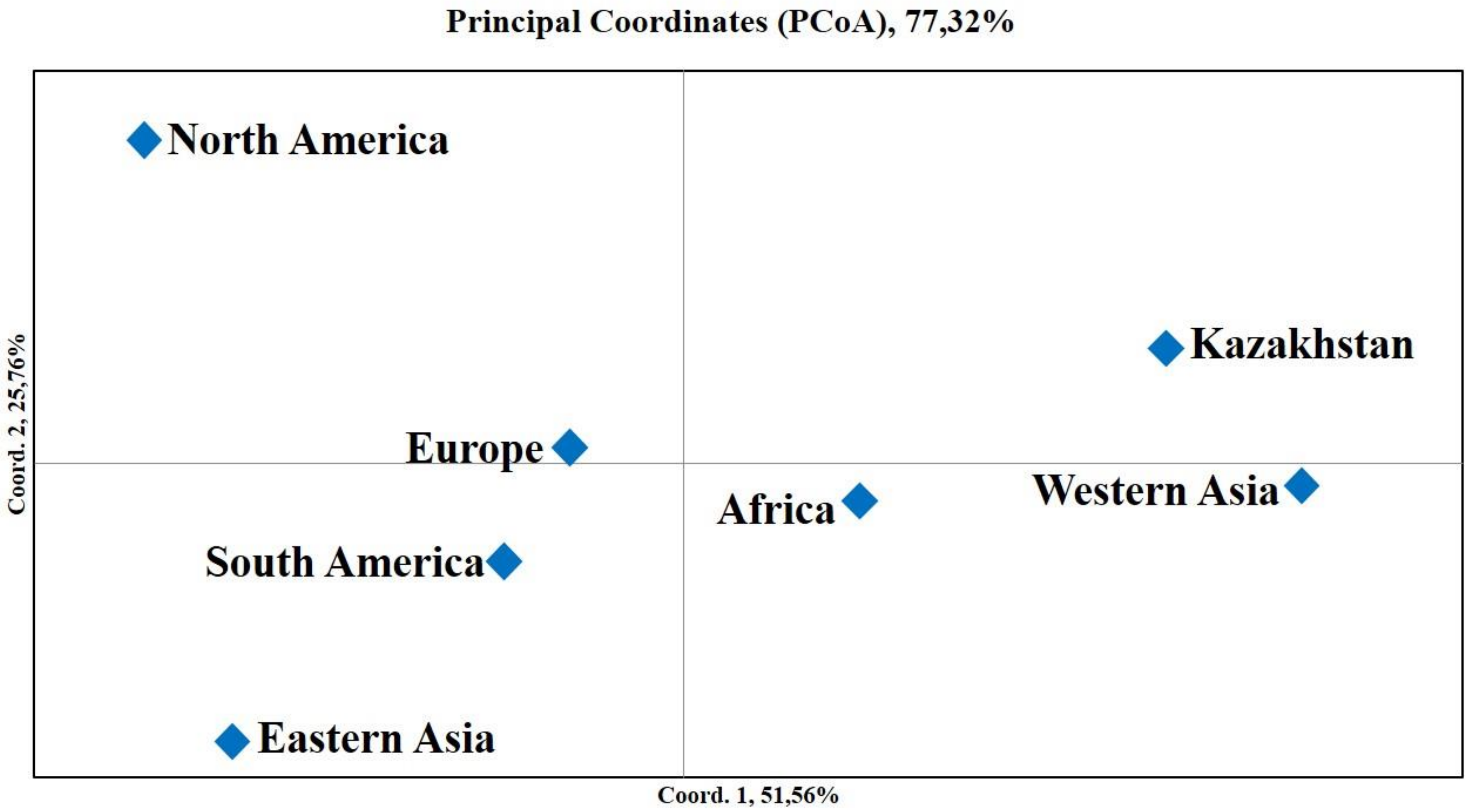

2R barley, 3H

597 samples (7 groups),
293 SNP

Principal Coordinates Analysis (PCoA)

Pairwise Population Matrix of **Nei Unbiased Genetic Distance**

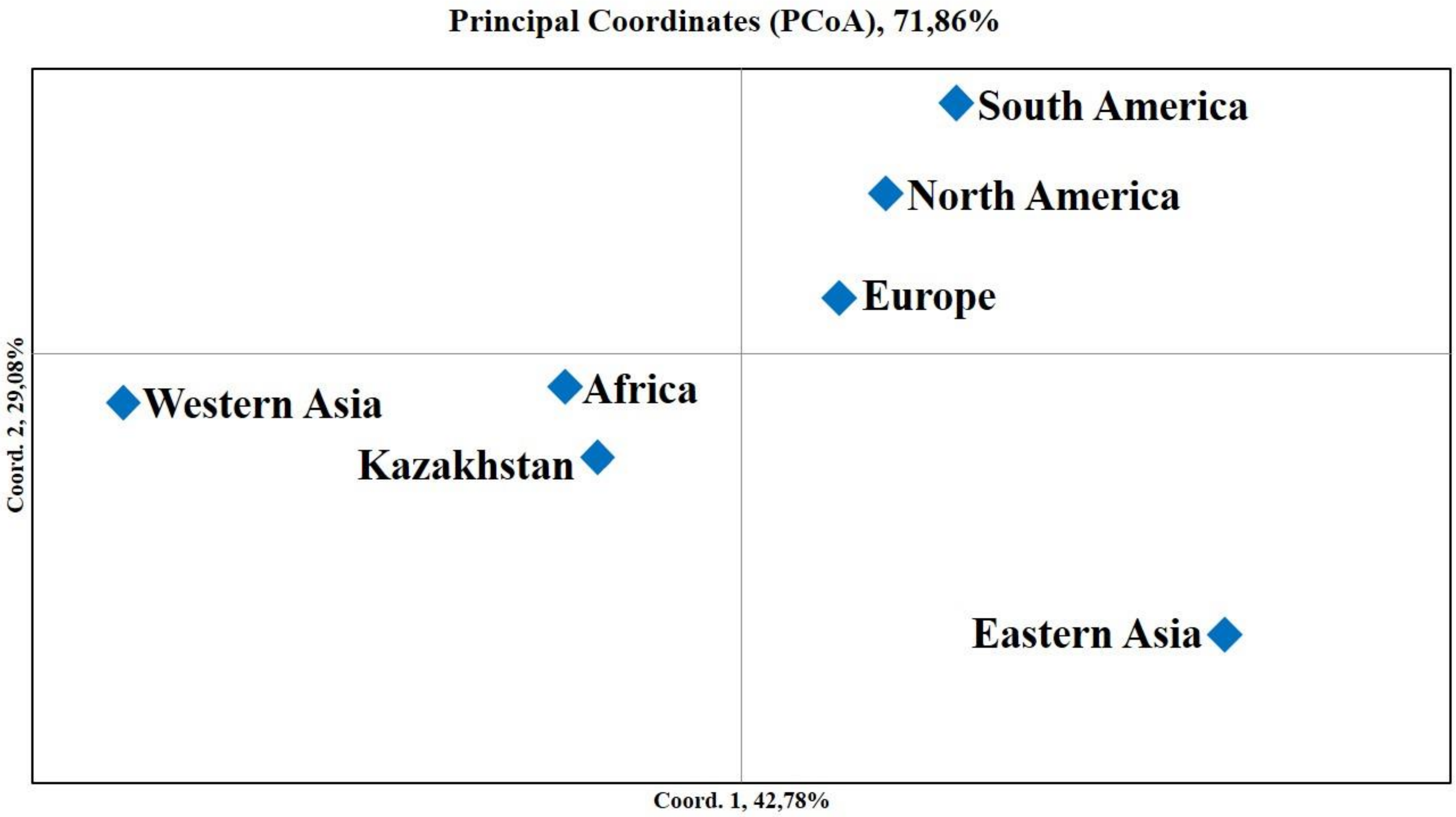

2R barley, 4H

Principal Coordinates Analysis (PCoA)

Pairwise Population Matrix of **Nei Unbiased Genetic Distance**

597 samples (7 groups),
230 SNP

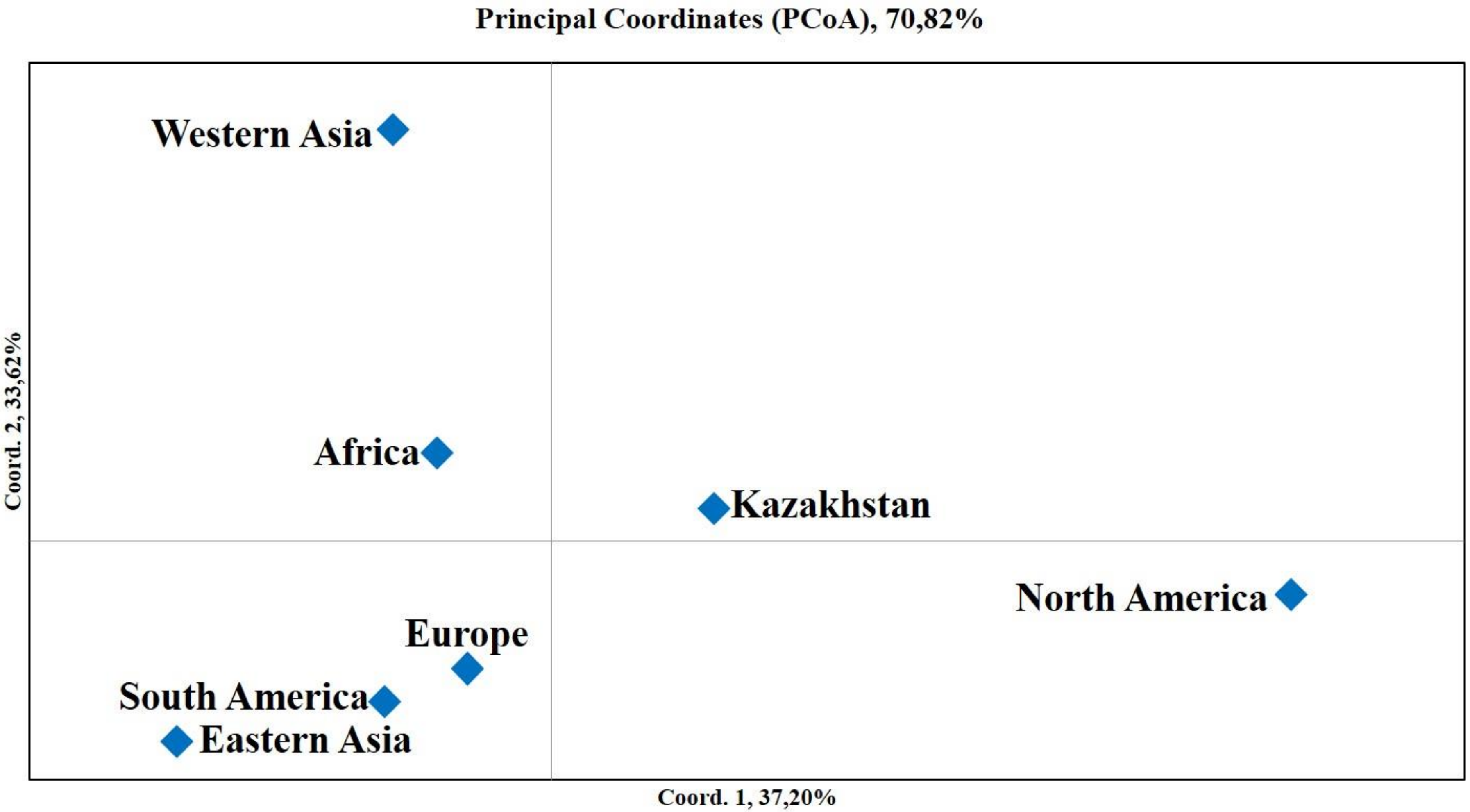

2R barley, 5H

597 samples (7 groups),
357 SNP

Principal Coordinates Analysis (PCoA)

Pairwise Population Matrix of **Nei Unbiased Genetic Distance**

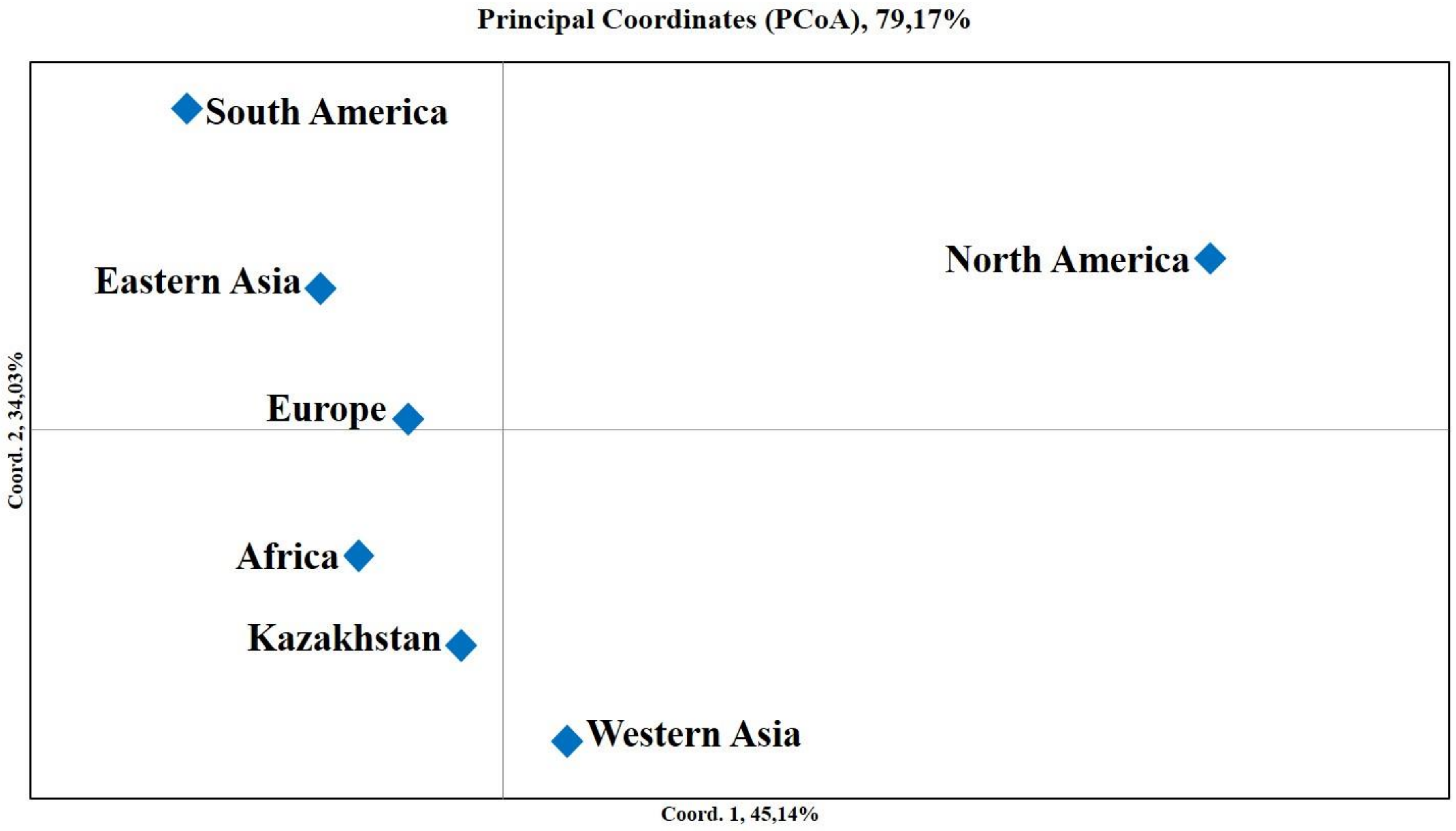

2R barley, 6H

597 samples (7 groups),
246 SNP

Principal Coordinates Analysis (PCoA)

Pairwise Population Matrix of **Nei Unbiased Genetic Distance** **(JPG)**

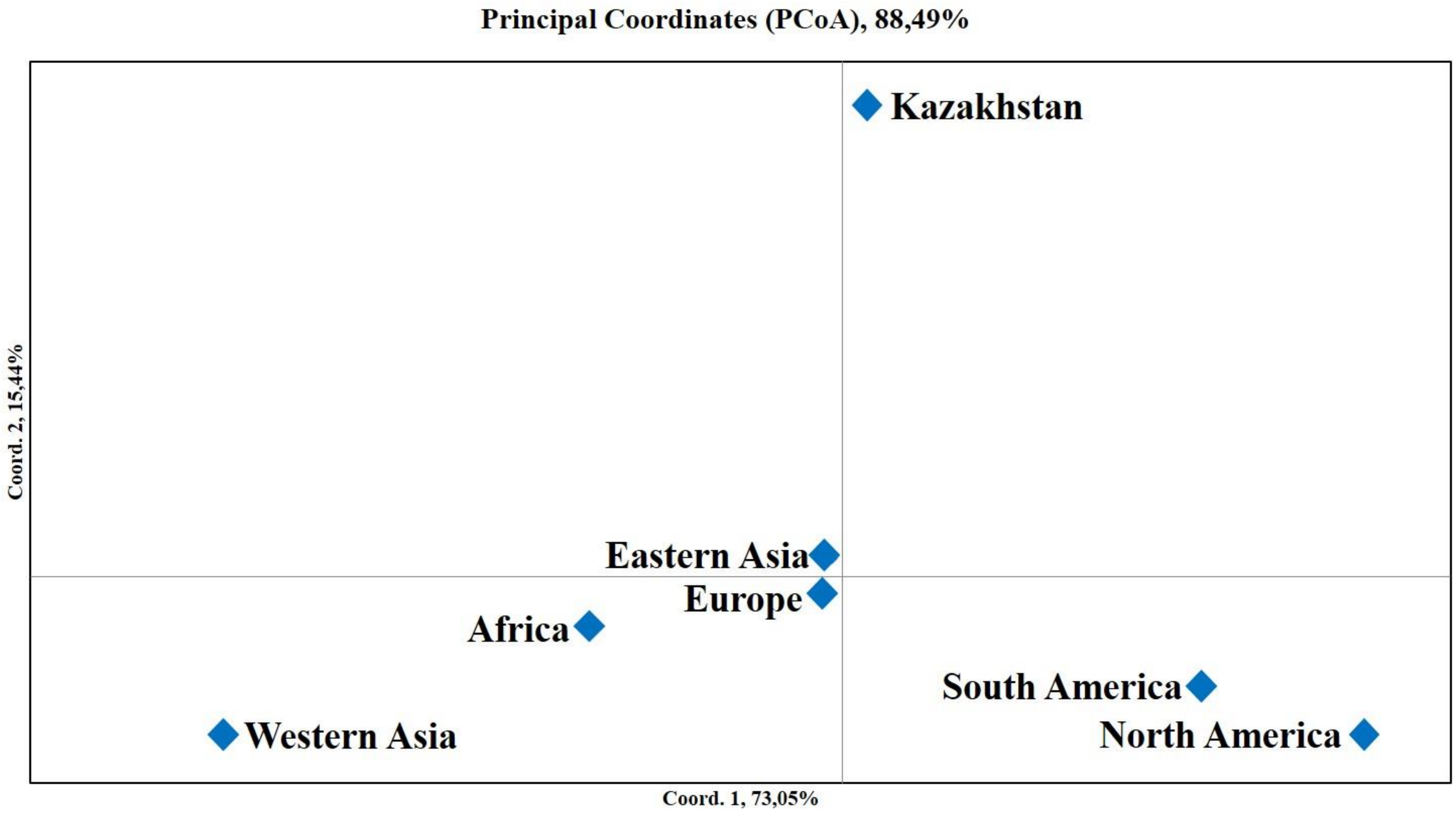

2R barley, 7H

597 samples (7 groups),
271 SNP

Principal Coordinates Analysis (PCoA)

Pairwise Population Matrix of **Nei Unbiased Genetic Distance**

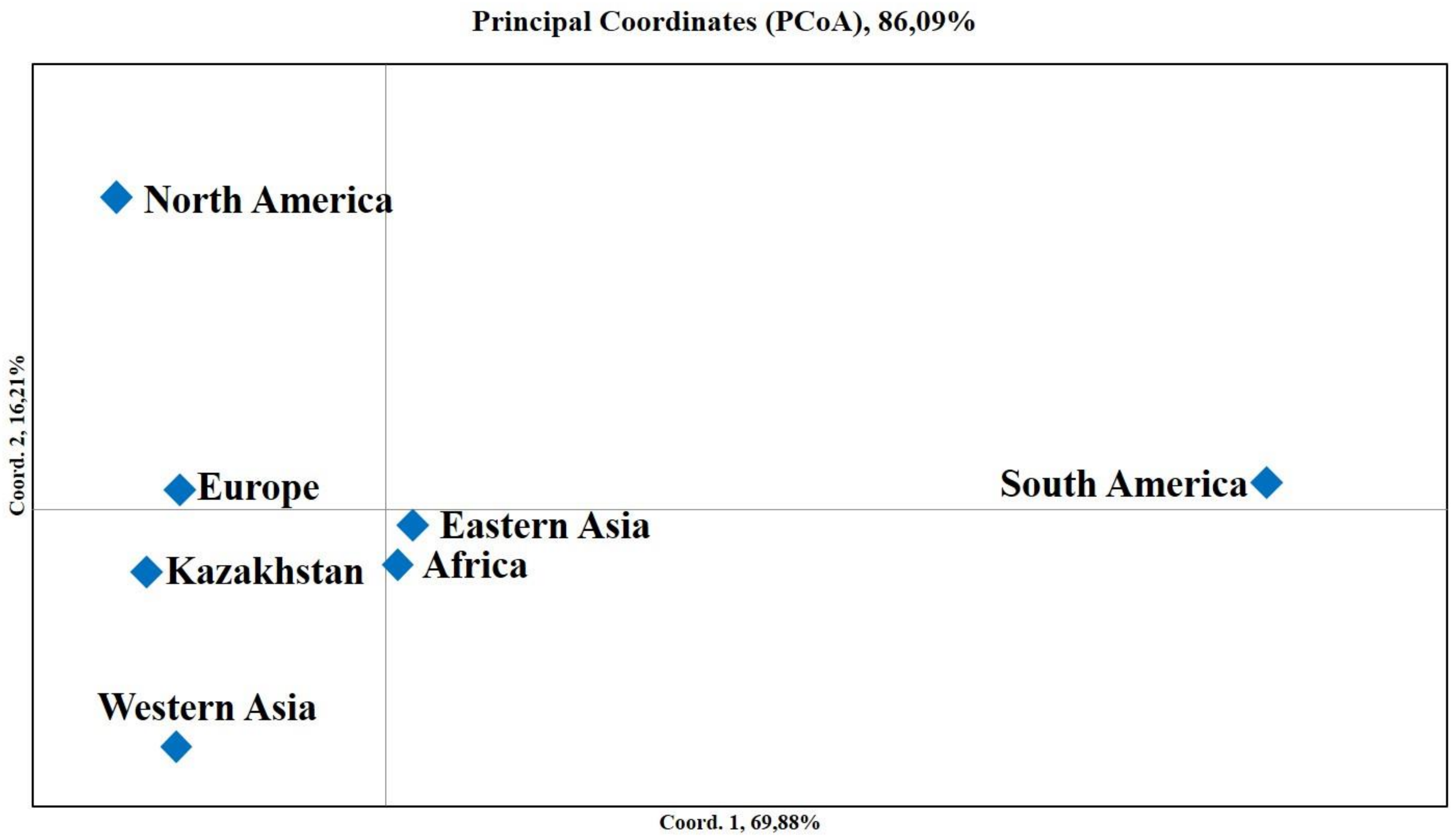

Supplement: Supplementary file 1 [file plants-10-02025-s001.zip › Figure S2.pdf]
