# Supplementary material for: Population Structure and Genetic Diversity of Two-Rowed Barley Accessions from Kazakhstan Based on SNP Genotyping Data
Source: Plants (Basel). 2021 Sep 27;10(10):2025. doi: 10.3390/plants10102025 (PMC8540147; doi:10.3390/plants10102025)
Supplement: Supplementary file 1 [file plants-10-02025-s001.zip › Figure S3.pdf]

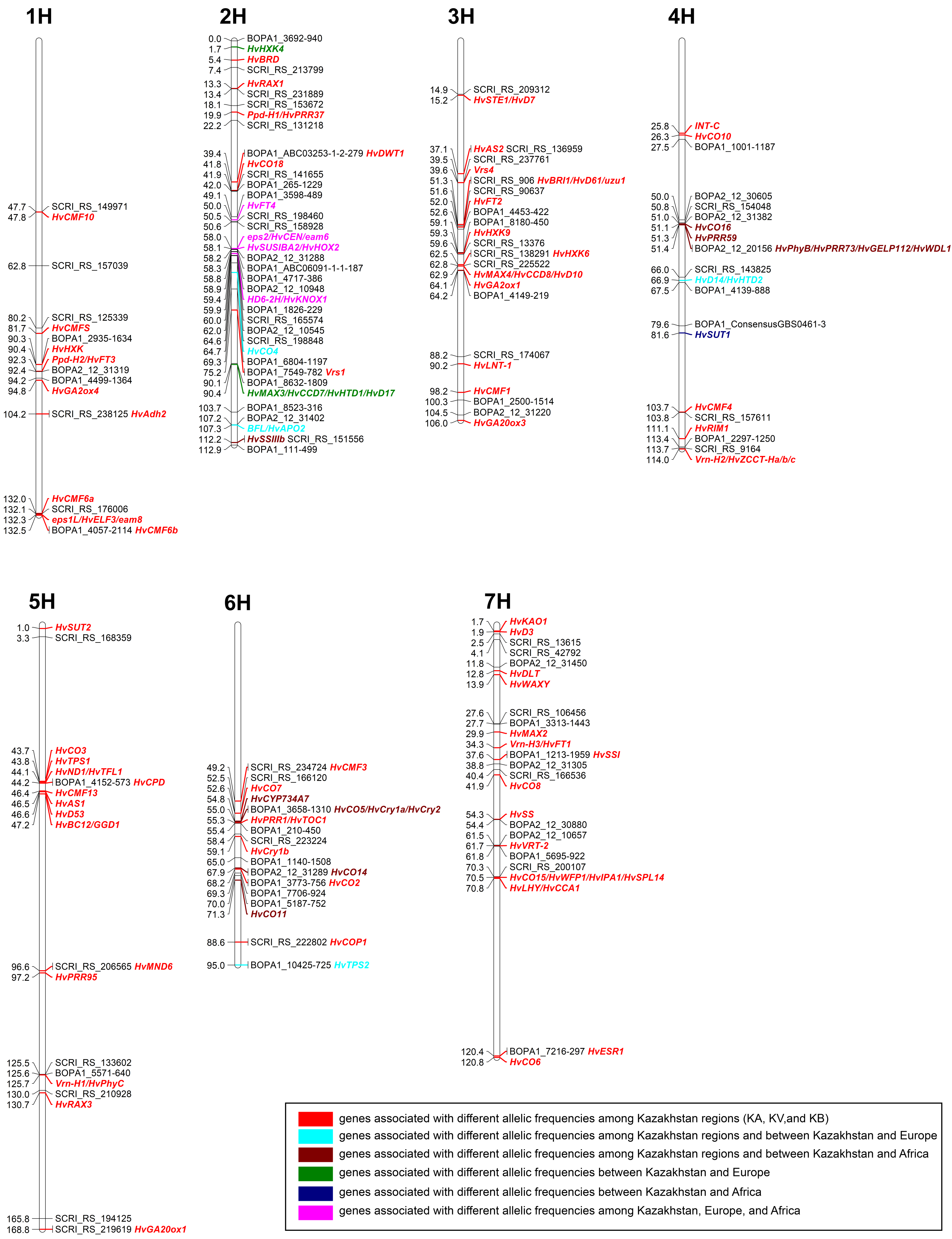
1H
47.7 SCRI_RS_149971
47.8 HvCMF10
62.8 SCRI_RS_157039
80.2 SCRI_RS_125339
81.7 HvCMFS
90.3 BOPA1_2935-1634
90.4 HvHXK
92.3 Ppd-H2/HvFT3
92.4 BOPA2_12_31319
94.2 BOPA1_4499-1364
94.8 HvGA2ox4
104.2 SCRI_RS_238125 HvAdh2
132.0 HvCMF6a
132.1 SCRI_RS_176006
132.3 eps1L/HvELF3/eam8
132.5 BOPA1_4057-2114 HvCMF6b
2H
0.0 BOPA1_3692-940
1.7 HvHXK4
5.4 HvBRD
7.4 SCRI_RS_213799
13.3 HvRAX1
13.4 SCRI_RS_231889
18.1 SCRI_RS_153672
19.9 Ppd-H1/HvPRR37
22.2 SCRI_RS_131218
39.4 BOPA1_ABC03253-1-2-279 HvDWT1
41.8 HvCO18
41.9 SCRI_RS_141655
42.0 BOPA1_265-1229
49.1 BOPA1_3598-489
50.0 HvFT4
50.5 SCRI_RS_198460
50.6 SCRI_RS_158928
58.0 eps2/HvCEN/eam6
58.1 HvSUSIBA2/HvHOX2
58.2 BOPA2_12_31288
58.3 BOPA1_ABC06091-1-1-187
58.8 BOPA1_4717-386
58.9 BOPA2_12_10948
59.4 HD6-2H/HvKNOX1
59.9 BOPA1_1826-229
60.0 SCRI_RS_165574
62.0 BOPA2_12_10545
64.6 SCRI_RS_198848
64.7 HvCO4
69.3 BOPA1_6804-1197
75.2 BOPA1_7549-782 Vrs1
90.1 BOPA1_8632-1809
90.4 HvMAX3/HvCCD7/HvHTD1/HvD17
103.7 BOPA1_8523-316
107.2 BOPA2_12_31402
107.3 BFL/HvAPO2
112.2 HvSSIIIb SCRI_RS_151556
112.9 BOPA1_111-499
3H
14.9 SCRI_RS_209312
15.2 HvSTE1/HvD7
37.1 HvAS2 SCRI_RS_136959
39.5 SCRI_RS_237761
39.6 Vrs4
51.3 SCRI_RS_906 HvBRI1/HvD61/uzu1
51.6 SCRI_RS_90637
52.0 HvFT2
52.6 BOPA1_4453-422
59.1 BOPA1_8180-450
59.3 HvHXK9
59.6 SCRI_RS_13376
62.5 SCRI_RS_138291 HvHXK6
62.8 SCRI_RS_225522
62.9 HvMAX4/HvCCD8/HvD10
64.1 HvGA2ox1
64.2 BOPA1_4149-219
88.2 SCRI_RS_174067
90.2 HvLNT-1
98.2 HvCMF1
100.3 BOPA1_2500-1514
104.5 BOPA2_12_31220
106.0 HvGA20ox3
4H
25.8 INT-C
26.3 HvCO10
27.5 BOPA1_1001-1187
50.0 BOPA2_12_30605
50.8 SCRI_RS_154048
51.0 BOPA2_12_31382
51.1 HvCO16
51.3 HvPRR59
51.4 BOPA2_12_20156 HvPhyB/HvPRR73/HvGELP112/HvWDL1
66.0 SCRI_RS_143825
66.9 HvD14/HvHTD2
67.5 BOPA1_4139-888
79.6 BOPA1_ConsensusGBS0461-3
81.6 HvSUT1
103.7 HvCMF4
103.8 SCRI_RS_157611
111.1 HvRIM1
113.4 BOPA1_2297-1250
113.7 SCRI_RS_9164
114.0 Vrn-H2/HvZCCT-Ha/b/c
5H
1.0 HvSUT2
3.3 SCRI_RS_168359
43.7 HvCO3
43.8 HvTPS1
44.1 HvND1/HvTFL1
44.2 BOPA1_4152-573 HvCPD
46.4 HvCMF13
46.5 HvAS1
46.6 HvD53
47.2 HvBC12/GGD1
96.6 SCRI_RS_206565 HvMND6
97.2 HvPRR95
125.5 SCRI_RS_133602
125.6 BOPA1_5571-640
125.7 Vrn-H1/HvPhyC
130.0 SCRI_RS_210928
130.7 HvRAX3
165.8 SCRI_RS_194125
168.8 SCRI_RS_219619 HvGA20ox1
6H
49.2 SCRI_RS_234724 HvCMF3
52.5 SCRI_RS_166120
52.6 HvCO7
54.8 HvCYP734A7
55.0 BOPA1_3658-1310 HvCO5/HvCry1a/HvCry2
55.3 HvPRR1/HvTOC1
55.4 BOPA1_210-450
58.4 SCRI_RS_223224
59.1 HvCry1b
65.0 BOPA1_1140-1508
67.9 BOPA2_12_31289 HvCO14
68.2 BOPA1_3773-756 HvCO2
69.3 BOPA1_7706-924
70.0 BOPA1_5187-752
71.3 HvCO11
88.6 SCRI_RS_222802 HvCOP1
95.0 BOPA1_10425-725 HvTPS2
7H
1.7 HvKAO1
1.9 HvD3
2.5 SCRI_RS_13615
4.1 SCRI_RS_42792
11.8 BOPA2_12_31450
12.8 HvDLT
13.9 HvWAXY
27.6 SCRI_RS_106456
27.7 BOPA1_3313-1443
29.9 HvMAX2
34.3 Vrn-H3/HvFT1
37.6 BOPA1_1213-1959 HvSSI
38.8 BOPA2_12_31305
40.4 SCRI_RS_166536
41.9 HvCO8
54.3 HvSS
54.4 BOPA2_12_30880
61.5 BOPA2_12_10657
61.7 HvVRT-2
61.8 BOPA1_5695-922
70.3 SCRI_RS_200107
70.5 HvCO15/HvWFP1/HvIPA1/HvSPL14
70.8 HvLHY/HvCCA1
120.4 BOPA1_7216-297 HvESR1
120.8 HvCO6
genes associated with different allelic frequencies among Kazakhstan regions (KA, KV,and KB)
genes associated with different allelic frequencies among Kazakhstan regions and between Kazakhstan and Europe
genes associated with different allelic frequencies among Kazakhstan regions and between Kazakhstan and Africa
genes associated with different allelic frequencies between Kazakhstan and Europe
genes associated with different allelic frequencies between Kazakhstan and Africa
genes associated with different allelic frequencies among Kazakhstan, Europe, and Africa
