# Supplementary figures and images for: Population Structure and Genetic Diversity of Two-Rowed Barley Accessions from Kazakhstan Based on SNP Genotyping Data
Source: Plants (Basel). 2021 Sep 27;10(10):2025. doi: 10.3390/plants10102025 (PMC8540147; doi:10.3390/plants10102025)

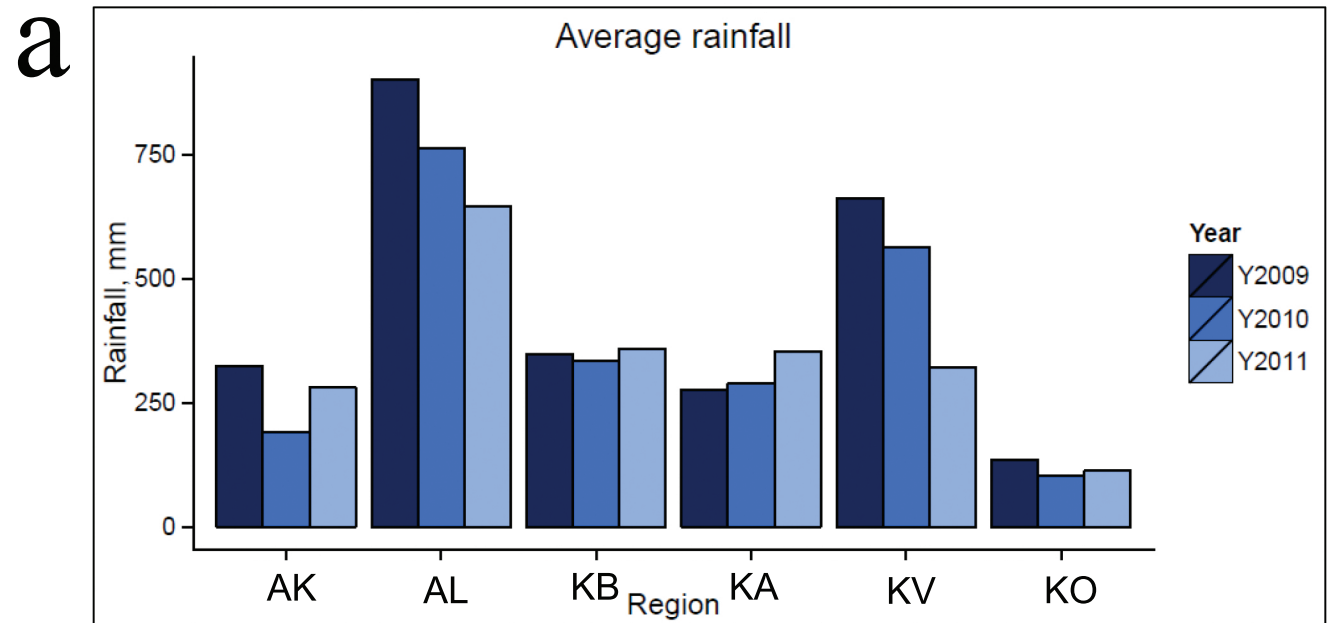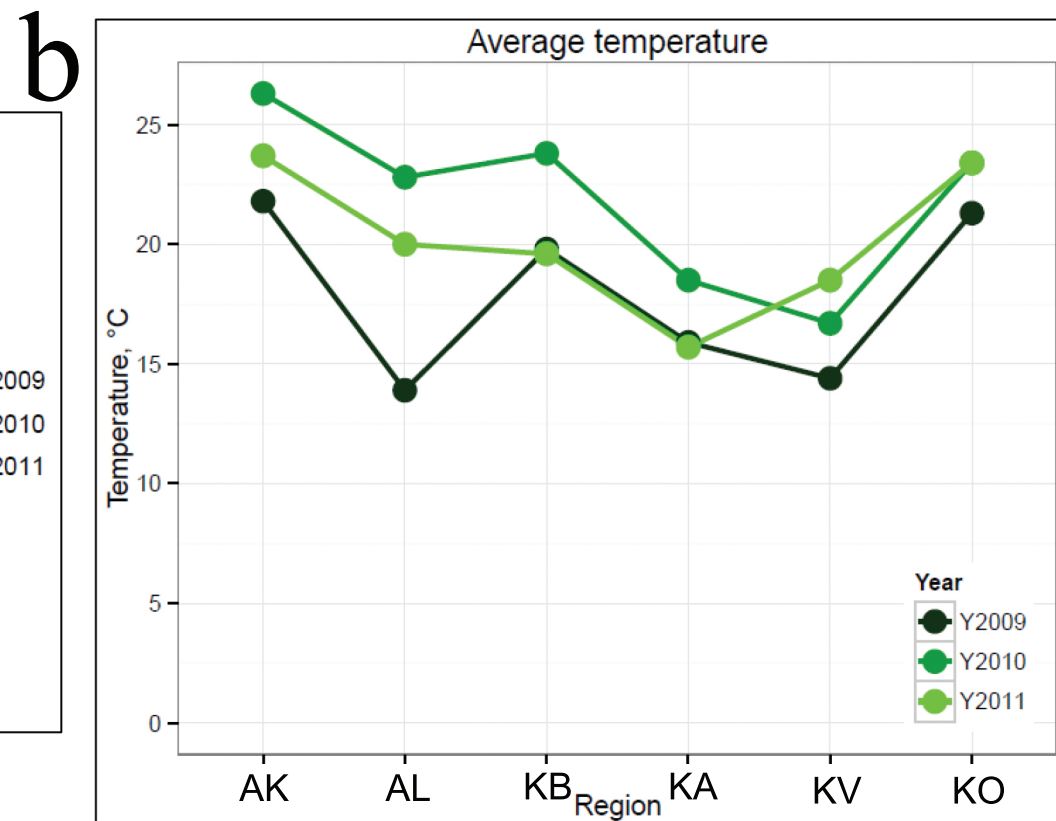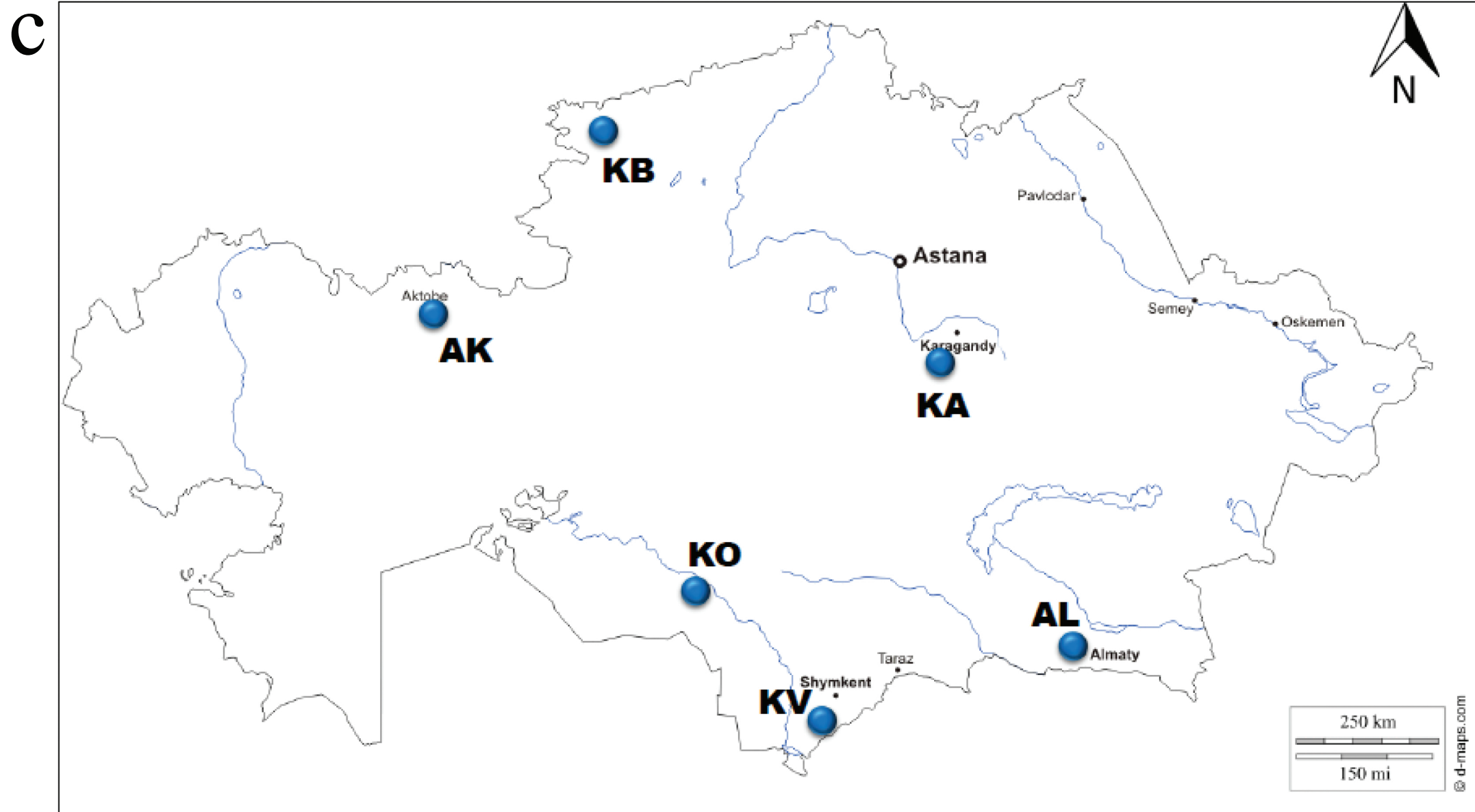

Supplement: Supplementary file 1 [file plants-10-02025-s001.zip › Figure S4.pdf]

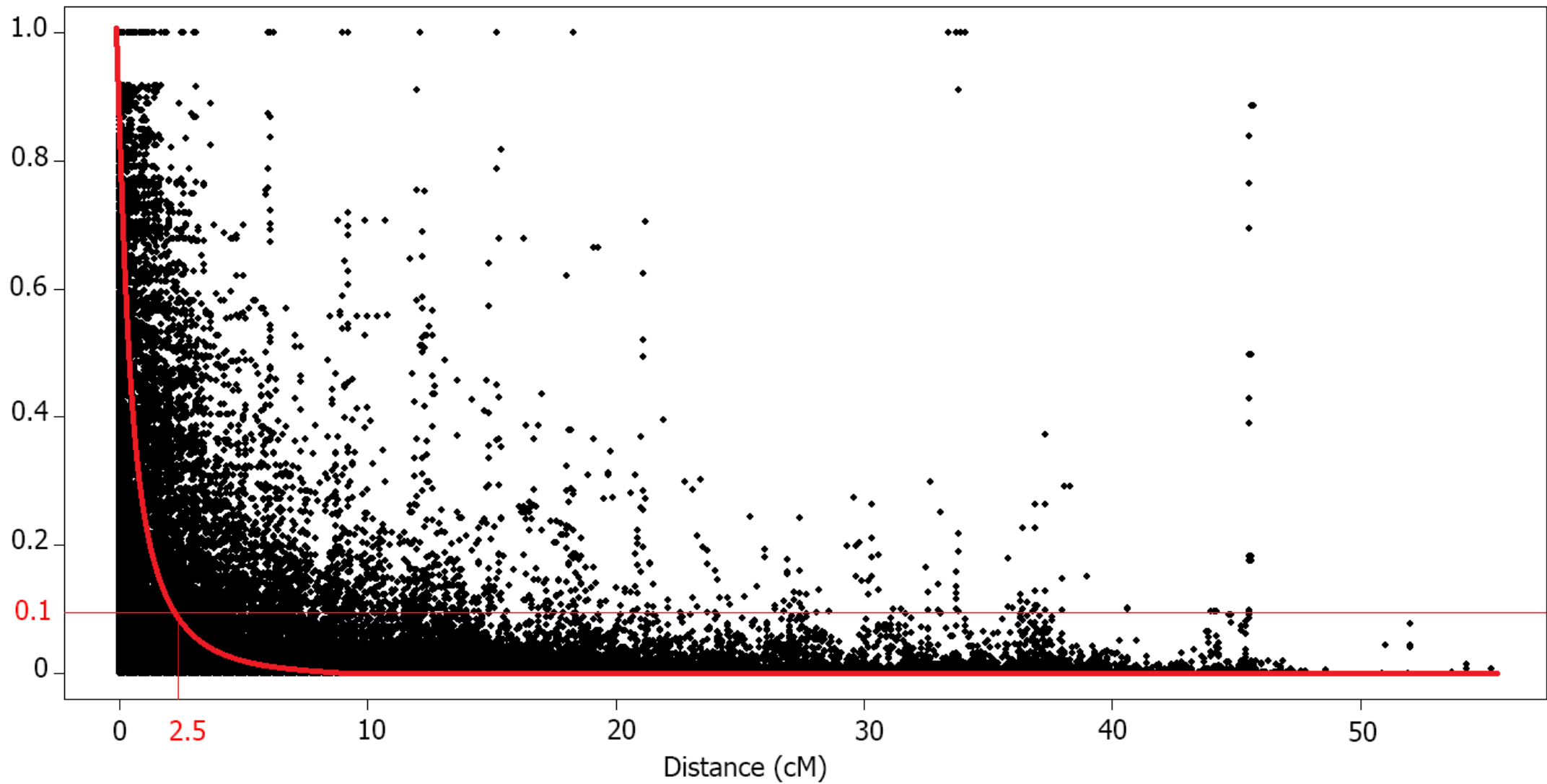

Supplement: Supplementary file 1 [file plants-10-02025-s001.zip › Figure S5.pdf]

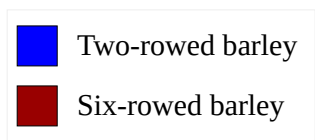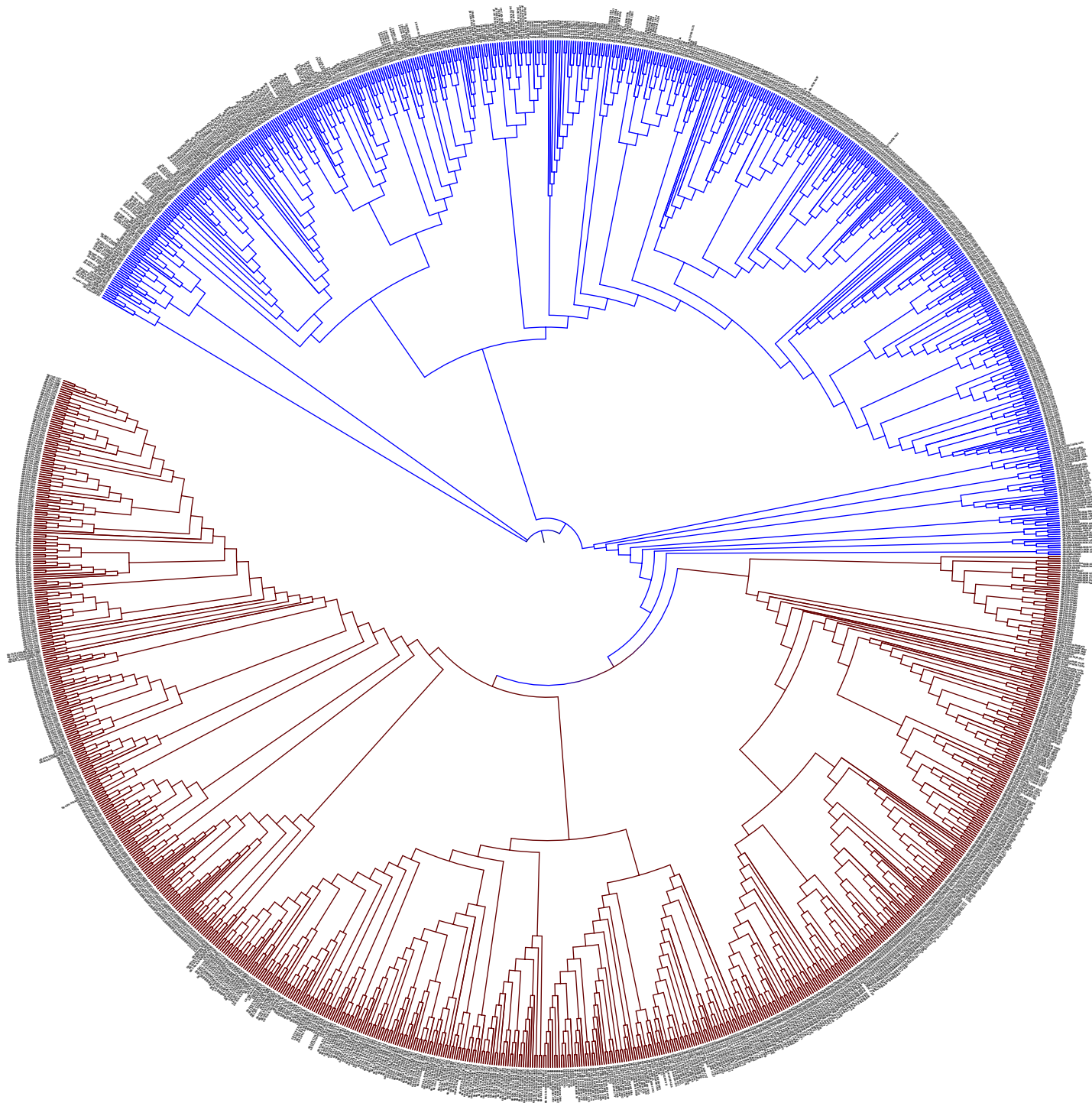

Supplement: Supplementary file 1 [file plants-10-02025-s001.zip › Figure S1.pdf]
